# Annealing‐Free Gradient Doping Strategy to Build Amorphous Nb:TiO_X_ Electron Transport Layer for Efficient Perovskite Solar Cells

**DOI:** 10.1002/smll.202510319

**Published:** 2025-11-06

**Authors:** Fang Luo, Dongbo Zhang, Shaozheng Chen, Yewon Lee, Jun‐Hyeok Kang, Joon Jang, Hyun‐Jung Jung, Seungmin Lee, Ji‐Yoon Chae, Jin‐Wook Lee, Han‐Ki Kim

**Affiliations:** ^1^ School of Advanced Materials Science and Engineering Sungkyunkwan University Suwon Gyunggi‐do 16419 Republic of Korea; ^2^ SKKU Advanced Institute of Nanotechnology Sungkyunkwan University Suwon Gyunggi‐do 16419 Republic of Korea; ^3^ Department of Display Engineering Sungkyunkwan University Suwon Gyunggi‐do 16419 Republic of Korea

**Keywords:** amorphous Nb:TiO_x_, annealing‐free, electron transport layer, gradient doping, perovskite solar cells

## Abstract

A low‐temperature, annealing‐free strategy is reported for fabricating Nb‐doped TiO_2_ (NTO) electron transport layers (ETLs) via dynamic co‐sputtering with controlled power modulation. This method produces a vertically graded composition (Ti‐rich at the bottom and Nb‐rich at the top) —without requiring any post‐deposition thermal treatment. The resulting NTO films exhibit a smooth, amorphous morphology and a chemically homogeneous interface. The engineered doping gradient enables deliberate modulation of the NTO band structure, optimizing energy level alignment with the perovskite absorber, mitigating interfacial energy barriers, and facilitating more efficient charge extraction. Despite the absence of annealing, the film exhibits low defect density and smooth interfaces, thereby minimizing recombination losses and improving open‐circuit voltage and fill factor. Perovskite solar cells (PSCs) incorporating the compositionally graded NTO ETLs achieve a power conversion efficiency of 19.9% and an open‐circuit voltage of 1.12 V, outperforming devices employing single‐target sputtered and conventional co‐sputtered ETLs. This work establishes a scalable and thermally benign pathway toward high‐performance, flexible, and cost‐effective PSCs.

## Graphical abstract

Compared with conventional single‐target or co‐sputtering methods, dynamic gradient co‐sputtering creates a vertically gradient Nb‐doped TiO_2_ (G‐NTO) electron transport layer in perovskite solar cells, improving electron extraction and interface stability. Notably, the G‐NTO films exhibit improved band alignment and charge transport without annealing, simplifying the fabrication and enabling a high PCE of 19.9% in perovskite devices.

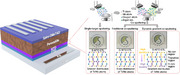

## 1 Introduction

Perovskite solar cells (PSCs) have rapidly emerged as a transformative photovoltaic technology, with certified power conversion efficiency (PCE) advancing from 3.8% to 27% in recent years, accompanied by significant improvements in operational stability.^[^ ^1^, ^2^, ^3^ ^]^ This exceptional progress is largely attributed to the development of optimized charge transport layers (CTLs), where both electron transport layers (ETLs) and hole transport layers (HTLs) play a crucial role in establishing selective contacts essential for efficient charge separation. Strategic advancements in material modification and interface engineering of these functional layers have enabled simultaneous enhancements in PCE and device stability, substantially advancing the commercial viability of PSCs.^[^ ^4^, ^5^, ^6^ ^]^ To meet the performance demands of state‐of‐the‐art PSCs, an ideal ETL must satisfy five key criteria: (i) high electron mobility for efficient charge extraction, (ii) appropriate energy level alignment for effective charge separation, (iii) robust chemical and thermal stability for long‐term operation, (iv) scalability, and (v) process compatibility for cost‐effective manufacturing.^[^ ^7^, ^8^, ^9^ ^]^ Among inorganic ETL candidates, titanium dioxide (TiO_2_) exhibits exceptional chemical stability, optimal band alignment with perovskite absorbers, and superior surface passivation capability.^[^ ^3^ ^]^ PSCs incorporating solution‐processed TiO_2_ ETLs have achieved certified PCEs exceeding 25%.^[^ ^10^, ^11^ ^]^ However, several intrinsic limitations persist: (1) ultraviolet‐induced degradation via oxygen desorption and Ti^3+^ defect formation, (2) high‐temperature processing requirements for crystalline phase formation, and (3) complex morphological control during fabrication—all of which impede cost‐effective scale‐up, particularly for flexible, roll‐to‐roll compatible substrates.^[^ ^3^, ^4^, ^12^, ^13^, ^14^ ^]^ These limitations necessitate innovative ETL design strategies that simultaneously address performance and stability challenges.

The photovoltaic efficiency of PSCs is predominantly governed by the effective extraction and transport of photogenerated charges through the CTLs to the electrodes.^[^ ^9^, ^15^ ^]^ This highlights the crucial importance of precisely engineering the perovskite/CTL interface to minimize defect states.^[^ ^16^, ^17^, ^18^ ^]^ Niobium pentoxide (Nb_2_O_5_) has demonstrated outstanding dielectric properties that suppress carrier recombination, enhancing open‐circuit voltage (*V*_OC_) and fill factor (FF) in PSCs.^[^ ^19^, ^20^, ^21^ ^]^ Substitutional doping of Ti^4+^ (0.605 Å) with Nb^5+^ (0.64 Å) introduces favorable modifications in the band structure, energy level alignment, charge transport behavior, and defect state distribution of Nb‐doped TiO_2_ (NTO) films, enhancing the overall efficiency and stability of PSCs.^[^ ^21^, ^22^, ^23^ ^]^ Despite its promise, solution processing,^[^ ^10^, ^24^, ^25^, ^26^, ^27^, ^28^ ^]^ the prevalent fabrication approach for ETLs, presents several technical limitations. These include (1) non‐uniform film morphology and high surface defect densities inherent to spin‐coating, (2) substantial precursor wastage during deposition,^[^ ^7^ ^]^ (3) formation of mixed‐valence Nb species (Nb^5+^ to Nb^1+^) that compromise device performance, and (4) limited scalability for large‐area deposition.^[^ ^7^, ^29^ ^]^ By contrast, magnetron sputtering has matured as a robust deposition method, offering decisive advantages for large‐area, low‐temperature fabrication of uniform NTO films with excellent reproducibility.^[^ ^30^, ^31^ ^]^ This vacuum‐based approach enables the deposition of pinhole‐free, ultrasmooth NTO films with precise nanostructural control via tunable gas dynamics while ensuring superior thickness uniformity and optimized optical properties.^[^ ^32^, ^33^ ^]^ Notably, the optoelectronic properties of sputtered NTO films can be systematically tuned by varying key deposition parameters, including working pressure, sputtering atmosphere, applied power, and film thickness.^[^ ^4^, ^31^, ^34^ ^]^

Currently, most reported studies utilize TiO_2_ films in various forms (dense, mesoporous, or nanorod arrays) as host matrices for Nb doping. However, such approaches often compromise the intrinsic properties of TiO_2_ owing to the nature of the doping process.^[^ ^7^, ^21^, ^35^, ^36^ ^]^ Moreover, inserting modified or passivated interlayers between the perovskite and CTL introduces additional heterojunction interfaces, generating potential energy barriers that hinder carrier transport.^[^ ^37^, ^38^ ^]^ Simultaneously, lattice mismatches at these heterointerfaces often introduce trap states, diminishing short‐circuit current density (*J*_SC_) and overall PCE. Intrinsic oxygen vacancies (*V*_O_) in metal oxide ETLs, coupled with suboptimal energy level alignment with perovskite layers, frequently result in charge recombination at the ETL/perovskite interface.^[^ ^38^, ^39^ ^]^ Consequently, effective defect passivation and precise energy level engineering at the interface are essential for minimizing energy losses and maximizing device performance. Gradient film engineering has emerged as an innovative strategy to address these challenges by introducing longitudinal compositional or structural gradients within the ETL. This methodology enables the concurrent optimization of charge transport pathways and the suppression of interfacial recombination through tailored energy level and defect state modulation.^[^ ^40^, ^41^ ^]^ Moreover, gradient architectures alleviate interfacial lattice mismatch and associated stress accumulation, enhancing mechanical robustness and long‐term operational reliability.^[^ ^42^ ^]^ Compared with conventional bilayer structures, co‐sputtering methods that facilitate gradual compositional variation within the film exhibit superior advantages in terms of interface homogeneity, structural coherence, device performance, and stability. Despite the potential of such approaches, systematic investigations into gradient NTO ETLs fabricated via co‐sputtering remain limited.^[^ ^37^ ^]^

In this study, we explore a compositionally graded NTO ETL fabricated via co‐sputtering of TiO_2_ and Nb_2_O_5_ targets, enabling fine‐tuned control over the Ti/Nb ratio to optimize energy level alignment, carrier mobility, and other key physicochemical properties. Compositional tuning enhanced interfacial compatibility and charge extraction efficiency, while gradient engineering reduced trap state densities and suppressed charge recombination. This represents a paradigm shift from basic film deposition to rational structural design. The resulting NTO films exhibit a uniform amorphous structure with minimized lattice mismatch‐induced defects. Furthermore, the multilayer homojunction architecture effectively modulates the band structure of the oxide semiconductor, promoting favorable energy level alignment, enhancing carrier transport and collection, and ultimately improving *J*_SC_ and overall PSC performance.^[^ ^43^ ^]^ Experimental validation confirmed theoretical predictions: the optimized gradient NTO ETL demonstrated reduced series resistance, increased electron mobility, and an ideal work function for PSCs. Most notably, the graded ETL achieved near‐perfect energy level alignment with the perovskite absorber, facilitating barrier‐free charge transport and yielding a champion PCE of 19.9% with a 1.12 V *V*_OC_. This work offers a versatile strategy for PSC optimization, integrating compositional precision with scalable fabrication, and represents a significant advance toward stable, efficient, and manufacturable perovskite photovoltaic technologies.

## 2 Results and Discussion

**Figure 1:**
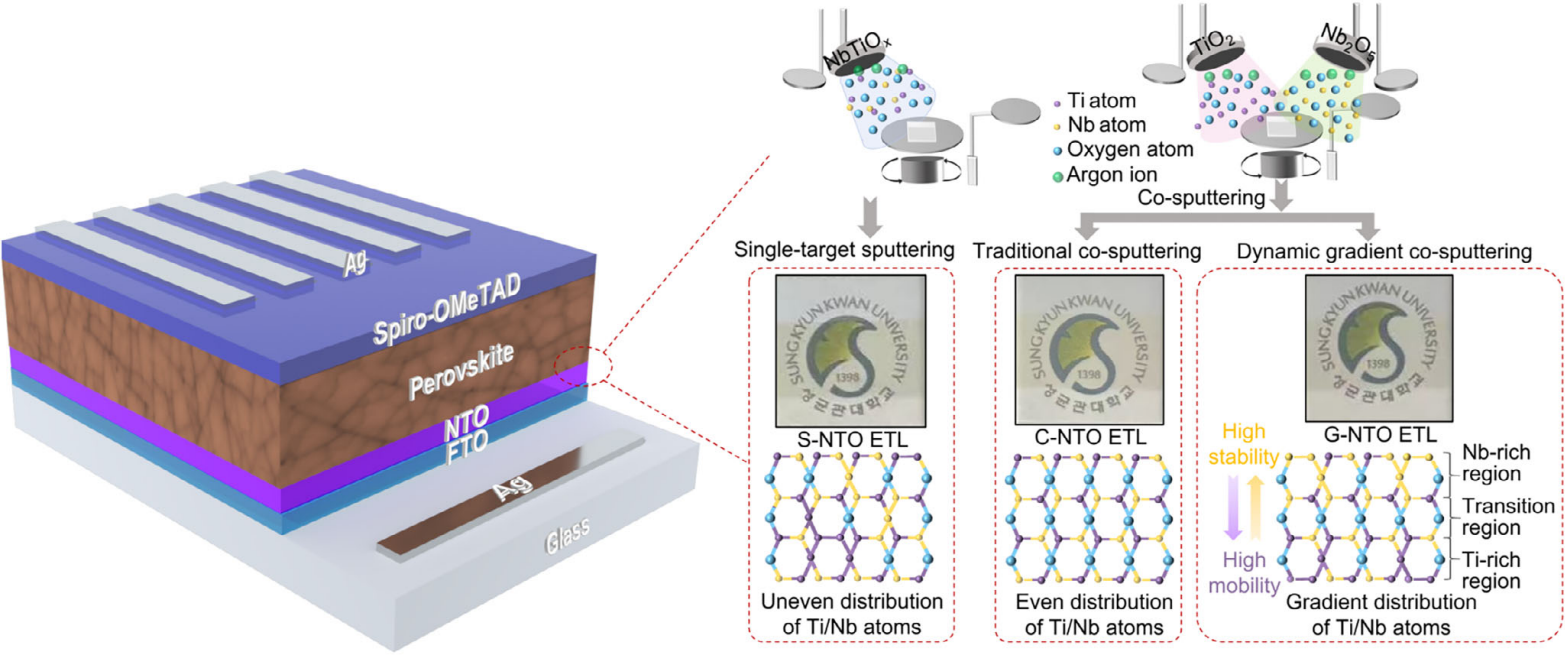
Schematic of PSCs incorporating different types of NTO ETLs prepared through three sputtering strategies: single‐target sputtering (S‐NTO), traditional co‐sputtering (C‐NTO), and dynamic gradient co‐sputtering (G‐NTO). The atomic distribution models demonstrate that dynamic co‐sputtering induces a vertical Ti/Nb compositional gradient from the Ti‐rich bottom region to the Nb‐rich top, enhancing charge mobility and stability.

To elucidate the impact of Nb‐Ti distribution within the ETL, we implemented three distinct sputtering methodologies: single‐target sputtering (S‐NTO), conventional co‐sputtering (C‐NTO), and dynamic gradient co‐sputtering (G‐NTO). As illustrated in **Figure** **1**, S‐NTO exhibits an irregular Ti/Nb atomic distribution, while C‐NTO yields a homogeneously mixed composition. By contrast, G‐NTO achieves a precisely modulated vertical compositional gradient, characterized by a Ti‐rich bottom layer and Nb‐rich surface. This gradient architecture is anticipated to synergistically enhance electron extraction, leveraging the high‐mobility Ti‐rich region, while simultaneously promoting device stability through the Nb‐rich upper layer. The corresponding device structure is shown in Figure S1 (Supporting Information). To optimize the fundamental sputtering conditions, we initially investigated the influence of deposition parameters (RF power, O_2_ gas flow, and working pressure) on the optical and electronic properties of S‐NTO thin films. The figure of merit (FoM = *T* ^10^/*R*_sheet_) was employed as a quantitative benchmark for identifying optimal deposition parameters.^[^ ^9^, ^34^ ^]^ In this expression, T^10^ represents the optical transmittance (typically measured at 550 nm), reflecting the transparency of the film, while R_sheet_ is the sheet resistance, representing the electrical conductivity. A higher FoM indicates a better trade‐off between optical transparency and electrical conductivity. Therefore, this parameter was employed as a benchmark to determine the optimal deposition conditions for achieving high‐quality transparent conductive films. In this study, the films were deposited on glass substrates for the FoM measurements. Systematic modulation of these variables revealed a strong dependence of film transmittance and electrical conductivity on the sputtering environment. Experimental optimization established the ideal conditions as an RF power of 100 W, a pure argon (Ar) atmosphere, and a working pressure of 4 mTorr (Figures S2 and S3, Supporting Information). Under these parameters, S‐NTO films exhibited a favorable balance between electron transport efficiency and optical transparency.

Elevated sputtering power beyond 100 W resulted in increased surface roughness, compromising optical performance and charge transport. Employing a pure Ar atmosphere effectively suppressed excessive oxidation and preserved an optimal concentration of *V*_O_ in the S‐NTO film, enhancing its electrical conductivity. By contrast, introducing O_2_ into the sputtering gas reduced the density of *V*_O_ and promoted the formation of over‐stoichiometric oxide phases, leading to diminished electronic performance. A working pressure of 4 mTorr was found to facilitate the growth of smooth, densely packed films, whereas lower pressures yielded porous morphologies that impeded electron mobility.

Figure S4 (Supporting Information) presents the contact angle measurements of S‐NTO films fabricated under various sputtering conditions. Under moderate power and pressure settings, the films exhibited the highest contact angles, likely attributed to optimized surface morphology and roughness that promoted hydrophobicity. However, following ultraviolet ozone treatment (UV OT), the contact angles for all S‐NTO films decreased to below 20°, indicating a substantial enhancement in surface wettability. This increased hydrophilicity is advantageous for promoting strong interfacial adhesion between the ETL and the subsequently deposited perovskite layer.

**Figure 2:**
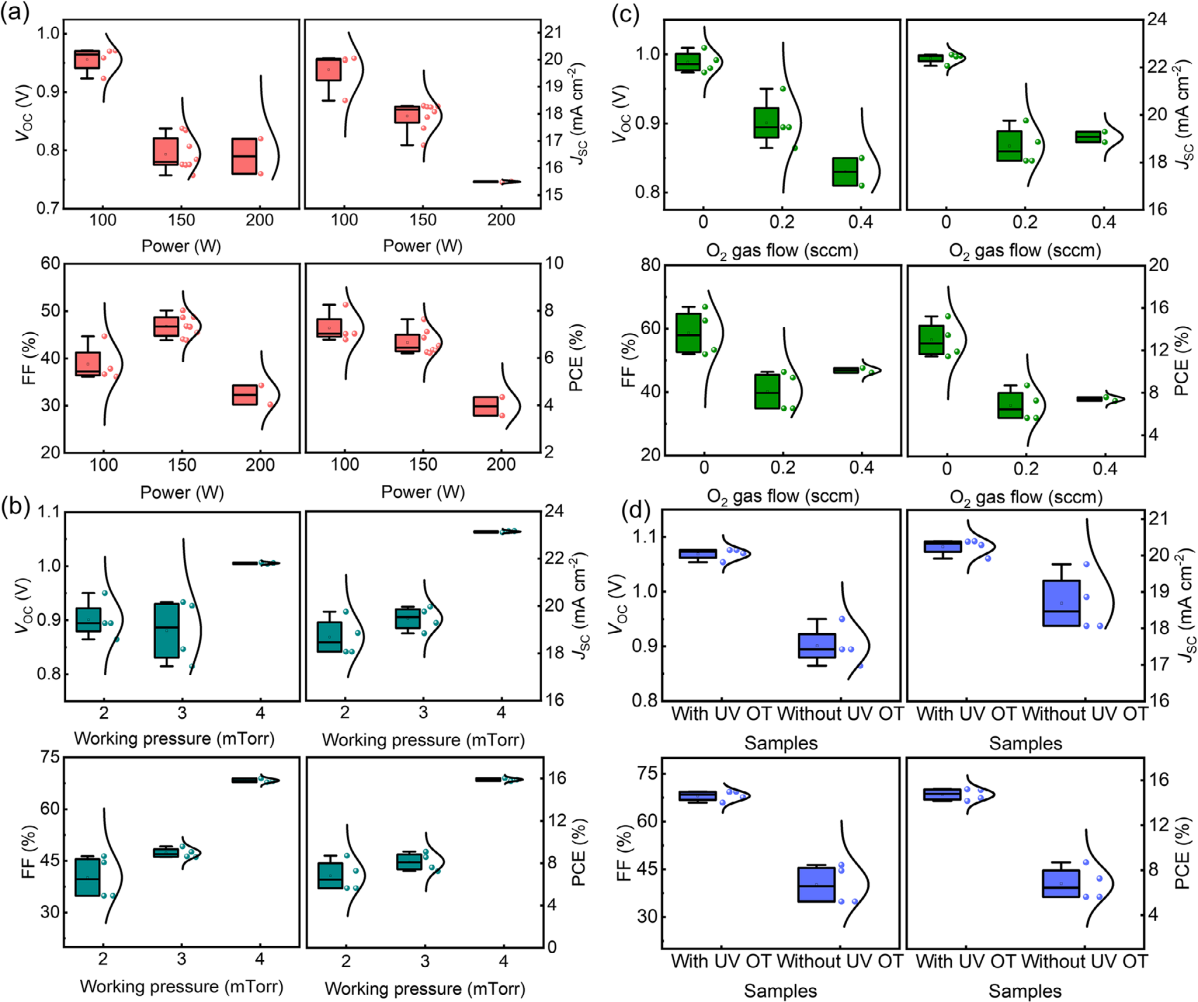
Photovoltaic performance of S‐NTO ETL‐based PSCs under different sputtering parameters such as a) RF powers, b) O_2_ gas flows, and c) working pressures. d) Comparison of photovoltaic performance of S‐NTO ETL‐based PSCs with and without UV ozone treatment.

**Figure** **2** comprehensively illustrates the impact of sputtering parameters and post‐treatment processes on the photovoltaic performance of PSCs employing S‐NTO as the ETL. For a preliminary comparison, only four devices were fabricated and tested under each condition. The effects of sputtering power, working pressure, oxygen flow rate, and UV OT were systematically examined by evaluating key device metrics, including *V*_OC_, PCE, *J*_SC_, and FF. As shown in Figure 2a, increasing the sputtering power from 100 to 200 W results in a progressive decline in device performance. At an optimal power of 100 W, the devices exhibit a relatively high *V*_OC_ of ≈1.0 V and a *J*_SC_ of ≈21 mA·cm^−2^, resulting in a PCE of ≈9%. Lower sputtering power is beneficial for improving film quality and device performance. However, excessively low power may result in insufficient deposition rate and poor film coverage, which can negatively affect the device characteristics.^[^ ^9^ ^]^ By contrast, devices sputtered at 200 W suffered significant reductions across all photovoltaic parameters, with PCE dropping below 5%. This degradation is attributed to plasma‐induced damage at elevated power levels, which compromises film integrity and interfacial contact quality, impeding charge transport and extraction. Figure 2b explores the influence of working pressure (2, 3, and 4 mTorr) on device performance. The optimal performance is observed at 4 mTorr, with a *V*_OC_ reaching 1.01 V, *J*_SC_ of up to 23 mA·cm^−2^, FF exceeding 70%, and PCE exceeding 16%. Higher working pressure can promote better film formation by reducing energetic particle bombardment. Nonetheless, excessively high pressure may cause excessive scattering of sputtered species, reducing their kinetic energy and deteriorating film density.^[^ ^44^ ^]^ Lower pressures (2 and 3 mTorr) likely impart excessive kinetic energy to sputtered species, resulting in interfacial damage and inferior charge transport characteristics. Figure 2c investigates the effect of introducing oxygen into the sputtering atmosphere. Counterintuitively, the highest device performance was achieved under a pure Ar environment (0 sccm O_2_), with *V*_OC_, PCE, *J*_SC_, and FF reaching optimal values, resulting in a maximum PCE of ≈16%. Increasing the oxygen flow rate to 0.2 and 0.4 sccm led to a notable decline in device performance. This suggests that even a small amount of oxygen introduced during the deposition process can cause excessive oxidation of the S‐NTO films. Such oxidation does not necessarily create additional oxygen vacancies but instead leads to a more stoichiometric or even over‐oxidized Ti^4+^/Nb^5+^ phase. This change can disturb the optimal balance between oxygen vacancies and carrier concentration, thereby reducing conductivity and mobility due to increased carrier scattering and suppressed electronic transport pathways.^[^ ^9^, ^45^ ^]^ In contrast, deposition under a pure Ar atmosphere results in a slightly oxygen‐deficient phase, where a moderate concentration of oxygen vacancies can beneficially enhance n‐type conductivity without introducing deep defect states.^[^ ^44^ ^]^ This phase exhibits higher intrinsic conductivity and fewer trap states, thereby facilitating more efficient electron transport and extraction in the device. Overall, careful control of the oxygen partial pressure during deposition is crucial to optimizing the electronic properties of S‐NTO films and achieving high device performance.

**Figure 3:**
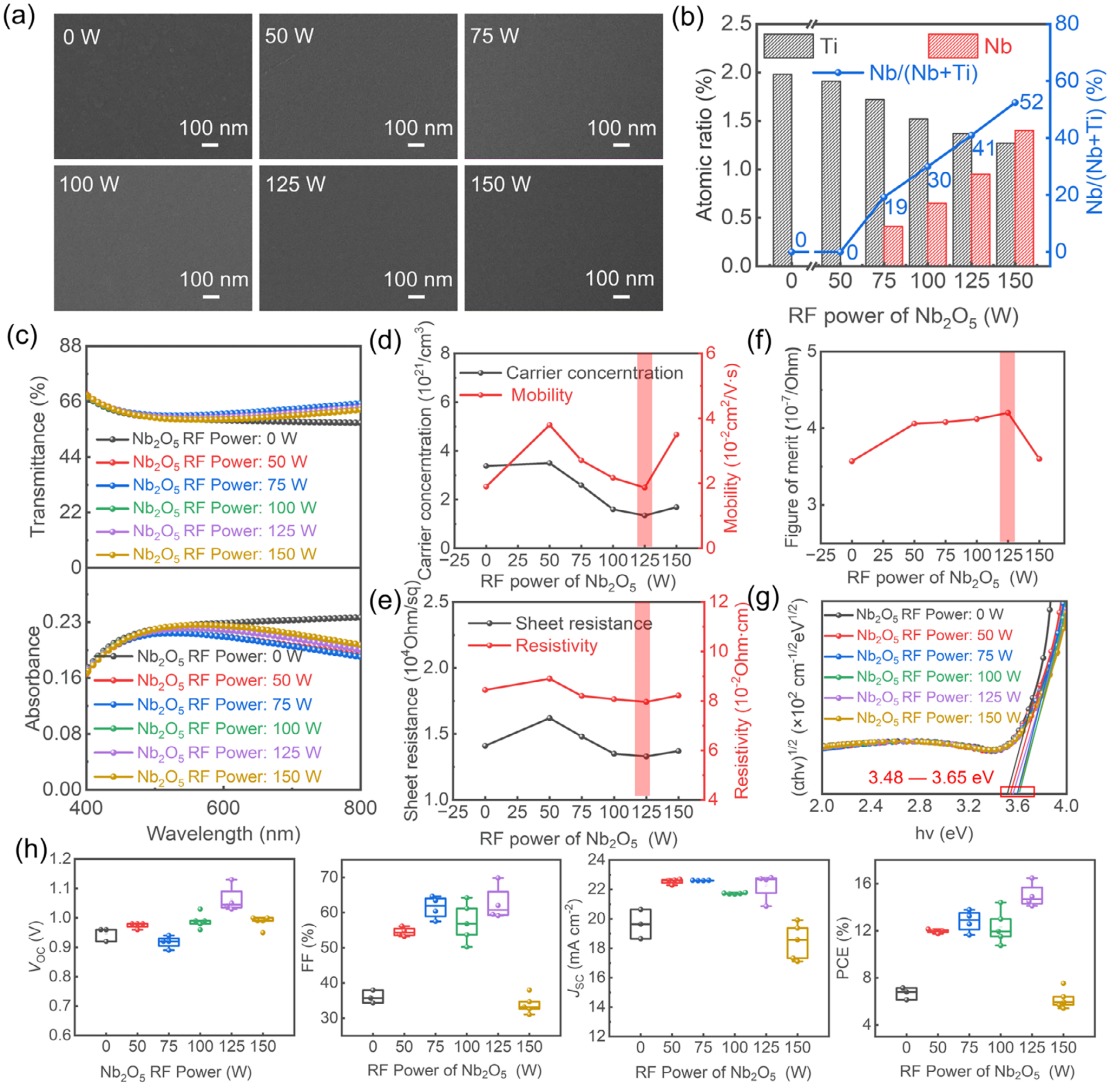
a) Surface SEM images of NTO films under different Nb_2_O_5_ RF powers. b) Atomic ratios of Ti and Nb and the relative ratio of Nb/(Ti+Nb) as functions of Nb_2_O_5_ RF powers. c) Optical properties of NTO films under different Nb_2_O_5_ RF powers. d,e) Hall measurement results of NTO films under different Nb_2_O_5_ RF powers. f) FoM values of NTO films under different Nb_2_O_5_ RF powers. g) Tauc's plot of NTO films under different Nb_2_O_5_ RF powers. h) Photovoltaic performance of C‐NTO ETL‐based PSCs according to RF power applied to the Nb_2_O_5_ target.

Figure 2d compares devices fabricated with and without UV OT. Untreated devices exhibit relatively poor performance, with a *V*_OC_, FF, and PCE of ≈0.9 V, 45%, and 8%, respectively. Following UV OT, all photovoltaic parameters are significantly improved: *V*_OC_ exceeds 1.05 V, FF surpasses 70%, and PCE increases to ≈16%. These improvements are attributed to the generation of active oxygen species during UV exposure, which facilitates defect passivation and further oxidation of the S‐NTO film, enhancing electronic properties and interfacial energy alignment. In summary, the observed performance trends of PSCs employing S‐NTO films closely aligned with the previously determined FoM values. Optimal device performance was achieved under the following sputtering conditions: 100 W of power, 4 mTorr of working pressure, and a pure argon atmosphere. Compared with single‐target sputtering, co‐sputtering allows the simultaneous use of multiple targets, offering enhanced compositional tunability and facilitating more precise control over the resulting film's chemical composition. This capability proves instrumental in tailoring the optical and electronic properties of the ETL. To establish a foundation for compositional engineering, we initially conducted a systematic optimization of sputtering parameters for the TiO_2_ target. By modulating sputtering power, working gas pressure, and oxygen flow rate, a series of TiO_2_ films was deposited and subsequently analyzed (Figures S5 and S6, Supporting Information). The FoM values, calculated from the optical transmittance and *R*_sheet_ of the film, were employed as key performance indicators. The results revealed that the optimal film quality was attained under a sputtering power of 200 W, a working pressure of 2 mTorr, and an oxygen flow rate of 0 sccm. Therefore, these conditions were selected as the baseline for subsequent co‐sputtering experiments. Maintaining the optimized parameters for the TiO_2_ target, we proceeded to systematically vary the sputtering power of the Nb_2_O_5_ target to control the Nb doping concentration and fabricate a series of C‐NTO films. As shown in the planar field emission scanning electron microscope (FE‐SEM) images (**Figure** **3**a), all C‐NTO films exhibit uniform, compact, and pinhole‐free morphologies on glass substrates, irrespective of the Nb content. Notably, the undoped TiO_2_ films demonstrate comparatively larger grain sizes, which progressively diminish with increasing Nb concentration. This grain refinement effect is attributed to the incorporation of Nb, which modulates film growth dynamics. Elemental compositions of the C‐NTO films were determined via energy‐dispersive X‐ray spectroscopy (EDS), with the corresponding spectra provided in Figure S7 (Supporting Information). The atomic percentages of Ti and Nb were plotted against the sputtering power applied to the Nb_2_O_5_ target (Figure 3b). At sputtering powers of 50, 75, 100, 125, and 150 W, the corresponding Nb atomic concentrations in the films were measured as 0, 0.41, 0.65, 0.96, and 1.4 at.%, respectively. It is worth noting that the atomic ratios of Ti and Nb appear very low. This is because the 60 nm‐thick film is very thin, allowing the electron beam to penetrate to the underlying glass substrate during EDS, which contributes a strong oxygen signal. Nevertheless, the relative proportions of Ti and Nb within the film can still be assessed. Notably, at 50 W, the Nb concentration was below the EDS detection threshold, indicating an insufficient doping level under those conditions. As the Nb_2_O_5_ sputtering power increased from 75 to 150 W, the relative Nb/(Nb + Ti) atomic ratio gradually increased from 19% to 52%, indicating a controllable compositional modulation via co‐sputtering. Extrapolating from this trend, we estimate that at a sputtering power of 50 W, the relative Nb/(Nb + Ti) ratio is ≈8%, corresponding to a Nb atomic content of ≈0.17 at.%. Following UV OT, the contact angles of the co‐sputtered C‐NTO films significantly decreased across all Nb concentrations compared with those of their single‐target sputtered counterparts (Table S1, Supporting Information), indicating enhanced surface wettability. This improvement is expected to facilitate better interfacial adhesion between the ETL and perovskite layer. Optical characterization revealed that moderate Nb incorporation improved film transmittance. However, excessive Nb doping diminished optical transmittance (Figure 3c), suggesting the existence of an optimal doping range that balances optical clarity and electronic functionality.

As a wide‐bandgap semiconductor, TiO_2_ inherently exhibits low carrier concentrations, predominantly determined by thermally generated carriers or the presence of *V*_O_. With Nb doping, additional free carriers are introduced via the substitution of Ti^4+^ with Nb^5+^, which donates extra electrons to the conduction band, significantly enhancing the electrical conductivity of the film.^[^ ^34^ ^]^ As illustrated in Figure 3d, low levels of Nb doping significantly increase carrier concentration. However, with further doping, a decline is observed, attributed to the introduction of defect states and electron traps that hinder carrier transport. At even higher doping levels, the carrier concentration begins to rise again, presumably when the *V*_O_ concentration is restored to a threshold that compensates for the introduced trap states. Notably, the films fabricated with a Nb_2_O_5_ sputtering power of 125 W exhibit the lowest *R*_sheet_ and resistivity, along with the highest FoM values (Figure 3e,f), an optimal trade‐off between electrical conductivity and optical transmittance. Furthermore, Nb incorporation into the TiO_2_ lattice significantly alters the electronic structure of the thin film, leading to a widened bandgap (Figure 3g). This bandgap broadening can be attributed to the substitutional or interstitial incorporation of Nb atoms, which perturbs the local electronic environment and induces a shift in the conduction band edge.^[^ ^7^ ^]^ Solar simulation analysis of PSCs employing C‐NTO layers with various Nb concentrations (Figure 3h) revealed substantially enhanced photovoltaic performance in Nb‐doped devices compared with those based on pristine TiO_2_. Consistent with FoM evaluations, the highest device efficiency was achieved at a sputtering power of 125 W for the Nb_2_O_5_ target (n = 4 for each condition).

At low doping levels, the bandgap increase is primarily governed by the Burstein–Moss effect. However, at high doping levels, the formation of defect states and lattice disorder dominates, reducing the effective bandgap.^[^ ^46^ ^]^ While variations in carrier concentration and mobility may appear detrimental to charge transport, appropriate Nb doping effectively reduces interface trap densities, suppressing electron–hole recombination and enhancing the *V*_OC_. At low Nb doping levels, the weak electron extraction at the ETL/perovskite interface results in higher recombination rates and lower *V*_OC_. Conversely, optimal doping facilitates superior energy level alignment between the C‐NTO ETL and perovskite layer, reducing electron accumulation, improving injection efficiency, and consequently increasing *V*_OC_. Simultaneously, reductions in *R*_sheet_ enhance the *J*_SC_ by minimizing resistive losses and promoting more efficient charge collection. However, excessive Nb doping may lead to over‐conductivity, which undermines electron–hole selectivity, enhances interfacial recombination, and ultimately reduces *V*_OC_. Furthermore, the decrease in *R*_sheet_ also positively influences the FF, as it facilitates more efficient charge extraction. Nevertheless, ultra‐low *R*_sheet_ can induce adverse effects such as energy level misalignment and accelerated charge transport. These lead to space‐charge accumulation and increased non‐radiative recombination, negatively impacting *V*_OC_ and *J*_SC_. In addition, excessive doping may degrade optical transparency, reducing light absorption by the perovskite layer and limiting the overall PCE. The optimally doped C‐NTO films maintain a delicate balance between conductivity and transmittance, enhancing charge extraction efficiency and simultaneously improving *V*_OC_, *J*_SC_, FF, and PCE.

In contrast to single‐target and conventional co‐sputtering approaches, gradient co‐sputtering enables the formation of compositional and structural gradients within the NTO film, imparting synergistic enhancements to its optical, electronic, and stability‐related properties. In this technique, the sputtering power of each target is dynamically modulated during deposition to engineer a continuous vertical compositional gradient. Importantly, both targets remain active throughout the process, ensuring film homogeneity and avoiding abrupt energy barriers at compositional interfaces. In this study, Ti was preferentially enriched at the bottom of the film to promote efficient electron transport and favorable interfacial contact with the transparent electrode, while Nb was concentrated at the upper region of the film to enhance chemical stability and oxidation resistance. Two types of gradient configurations were designed to systematically investigate the effects of different compositional gradient profiles on photovoltaic performance.

**Figure 4:**
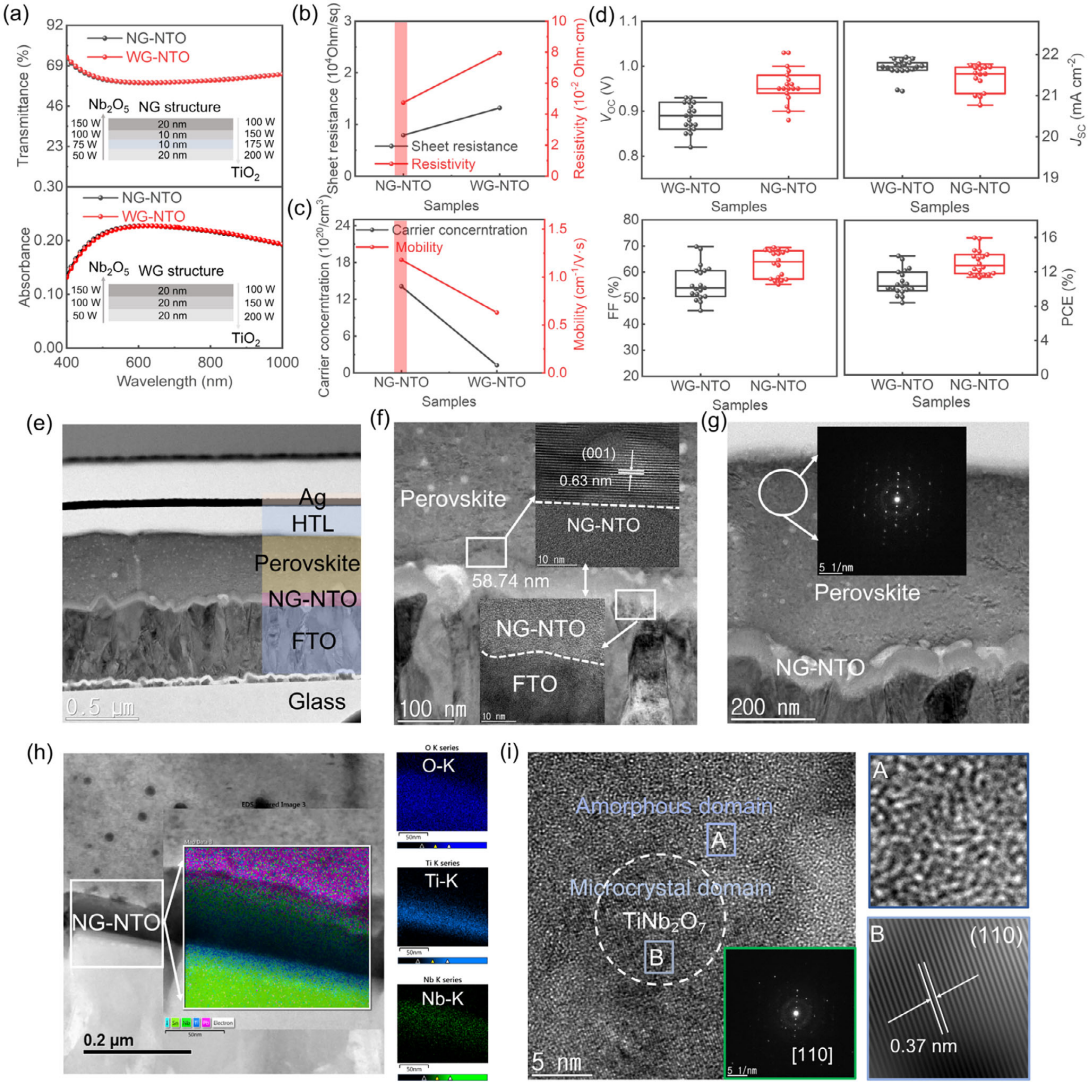
Comparison of NG‐NTO and WG‐NTO in PSC Devices. a) Optical properties of NG‐NTO and WG‐NTO with an inset of the schematic structure. b,c) Hall measurement results. d) Photovoltaic performance (*V*_OC_, *J*_SC_, FF, and PCE). e) Cross‐sectional transmission electron microscopy (TEM) image of device structure. f) Interface HRTEM images of perovskite/NG‐NTO and NG‐NTO/FTO. g) Selected area electron diffraction (SAED) pattern of the perovskite layer. h) Cross‐sectional scanning transmission electron microscopy‐energy dispersive X‐ray spectroscopy (STEM‐EDS) elemental mapping of the G‐NTO film, showing the depth distribution of O, Ti, and Nb elements. i) High‐resolution transmission electron microscopy (HRTEM) image showing the microcrystalline‐amorphous composite structure of the NG‐NTO thin film.

These two gradient‐layered structures, distinguished by the extent of compositional transition, were designated as narrow‐gradient NTO (NG‐NTO) and wide‐gradient NTO (WG‐NTO) films. The schematic insets in **Figure** **4**a illustrate the structural distinctions between the two. Notably, the transmittance and absorbance spectra of NG‐NTO and WG‐NTO films are nearly identical. However, the NG‐NTO films exhibit superior electrical characteristics, including higher carrier concentration and electron mobility, as well as lower *R*_sheet_ and resistivity (Figure 4b,c). As the number of gradient layers increases, the transitions between compositional regions become smoother, mitigating abrupt potential barriers within the film. This promotes enhanced carrier mobility through reduced scattering and interfacial resistance. Following UV OT, the NG‐NTO films also exhibit lower contact angles (Table S2, Supporting Information), indicating improved surface wettability—an essential attribute for ensuring strong interfacial adhesion with the perovskite layer.

PSCs utilizing ETLs with more finely graded compositional profiles demonstrate higher FoM values (Figure S8, Supporting Information) and enhanced photovoltaic performance (Figure 4d) (n = 15 for each condition). This can be attributed to improved energy‐level alignment and the suppression of interfacial charge recombination, facilitated by the gradient architecture at the ETL/perovskite interface. Figure 4e presents a cross‐sectional transmission electron microscopy (TEM) image of a complete device stack: Glass/FTO/NG‐NTO/Perovskite/HTL/Ag from bottom to top. The layers are well‐defined and compact, with no visible voids or interfacial delamination. A HRTEM image Figure 4f further reveals the interface between the NG‐NTO, perovskite layer, and FTO electrode. The NG‐NTO film thickness is at 58.74 nm, closely matching the designed specification of 60 nm, reflecting excellent deposition precision and process control. In Figure 4g, the SAED pattern of the NG‐NTO region shows weak and diffuse rings, consistent with a partially amorphous or low‐crystallinity phase. By contrast, the perovskite layer exhibits distinct polycrystalline diffraction rings and sharp diffraction spots. These indicate good crystallinity and preferential orientation, beneficial for efficient light absorption and charge transport. STEM‐EDS elemental mapping (Figure 4h) confirms the successful formation of a vertical compositional gradient within the NG‐NTO layer: Nb concentration gradually increases toward the bottom FTO interface, while Ti is enriched toward the top surface. This gradient facilitates optimized energy level alignment, promoting efficient electron extraction and transport. The moderate film thickness ensures the formation of an effective compositional gradient while maintaining sufficient charge conductivity and suppressing interfacial recombination—key factors contributing to enhanced device efficiency. Clear lattice fringes are observed in the perovskite region, with an interplanar spacing of ≈0.63 nm, corresponding to the (001) plane of the perovskite crystal, indicating a well‐ordered lattice. By contrast, the NG‐NTO layer exhibits no discernible lattice fringes, further confirming its amorphous or nanocrystalline nature. Both the perovskite above and the FTO below exhibit pronounced lattice planes (Figure S9, Supporting Information). As shown in Figure 4i, the NG‐NTO exhibits a microcrystalline–amorphous hybrid morphology. Importantly, despite the mixed‐phase structure, the interface between the NG‐NTO and the adjacent layers remains continuous and compact, with no observable interlayers or defects. The resulting uniformity and embedded doping gradient enable fine regulation of carrier dynamics, ensuring the requirements of electrical conductivity and energy level alignment are simultaneously met.

In conclusion, by engineering a continuous compositional gradient across the film thickness, different regions of the NG‐NTO layer are tailored to fulfill specific functional roles: the Ti‐rich bottom layer facilitates efficient electron transport, the middle region can be tuned for optimal light absorption via bandgap engineering, and the Nb‐rich top layer imparts superior chemical stability and oxidation resistance. This gradient architecture effectively reduces interfacial stress, improves charge carrier mobility, and contributes to higher PCE. Such multifunctional gradient films hold significant promise for integrating electron transport enhancement with interfacial passivation. However, the fabrication process becomes increasingly complex, necessitating precise control over sputtering parameters. Moreover, while introducing additional gradient layers may further improve device performance, it also raises potential concerns regarding interfacial instability and uneven charge transport, which could ultimately impact long‐term operational stability.

**Figure 5:**
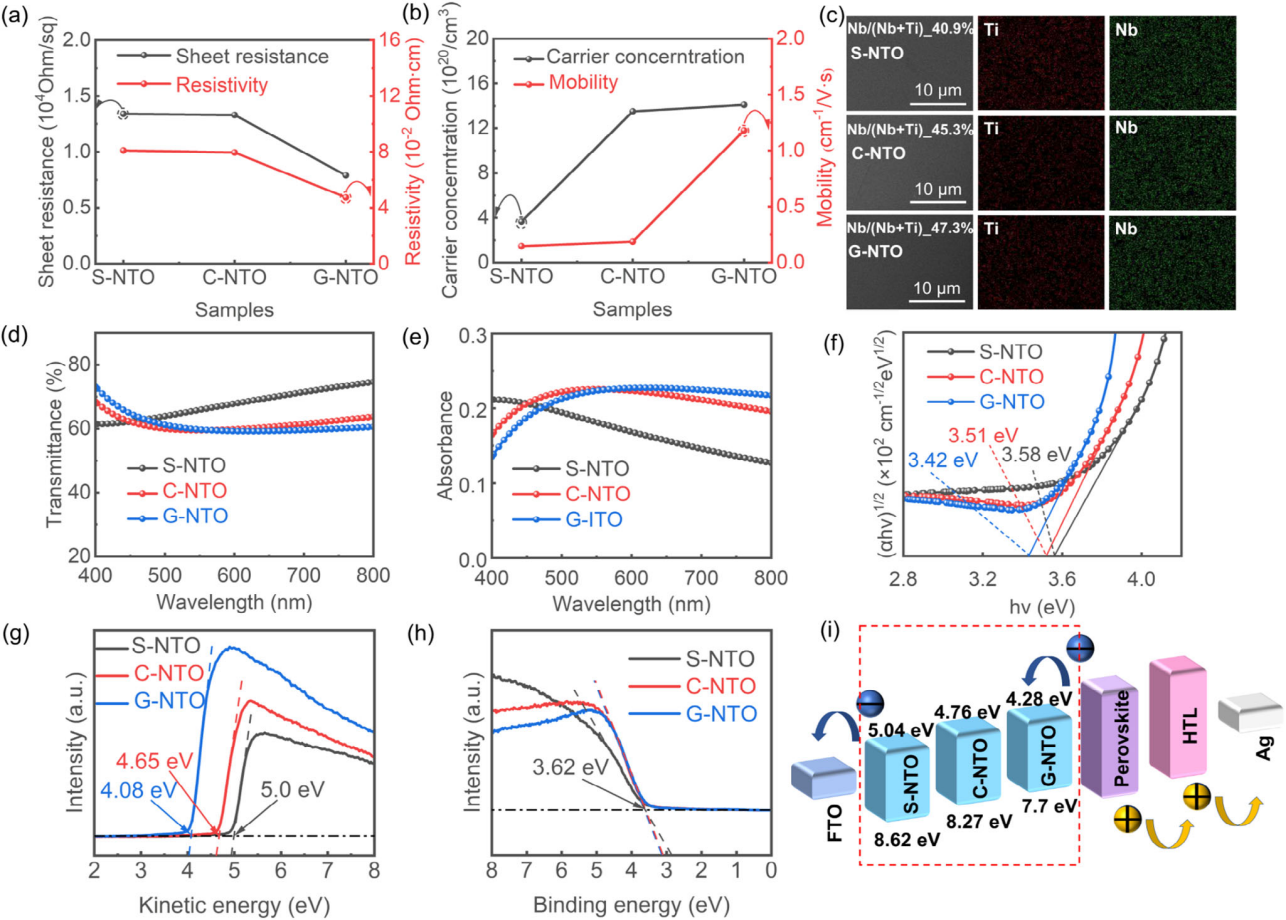
Comparison of S‐NTO, C‐NTO, and G‐NTO ETLs. a,b) Hall measurement results. c) SEM images and corresponding EDS elemental mappings. d,e) Optical properties. f) Tauc's plot is used to extract the optical bandgap values of different ETLs. g,h) Ultraviolet photoelectron spectroscopy (UPS) spectra for evaluating the work function and valence band maximum (VBM) positions. i) Schematic energy level diagrams of different ETLs, showing improved band alignment in G‐NTO for more efficient electron extraction.

Subsequently, we compared the optimized NTO films obtained via three different sputtering approaches. To evaluate the optoelectronic properties of different films, the films were deposited on glass substrates for UV–vis and Hall effect measurements. Hall measurements of the NTO films are shown in **Figure** **5**a,b. The G‐NTO films exhibit significantly reduced resistivity and *R*_sheet_, indicating enhanced electrical conductivity. By contrast, the S‐NTO and C‐NTO films exhibit relatively lower carrier mobilities, attributable to increased structural defects. The G‐NTO films demonstrate a more homogeneous composition, moderate carrier concentrations, and higher mobility. Such characteristics are attributable to reduced defect density and a tunable energy band structure. Increasing Nb doping enhances carrier concentration while maintaining a favorable optical transmittance, meeting the dual requirements for optoelectronic applications. Among the three film types, the G‐NTO films exhibit the highest carrier concentration and mobility, confirming superior electron transport characteristics. Nevertheless, these films exhibit the lowest optical transmittance, attributable to structural disorder, including amorphous or sub‐crystalline regions. This promotes light scattering and internal reflections, ultimately diminishing transparency. The NbTiO_x_ target employed had an approximate atomic ratio of 1:1. However, point EDS analysis revealed a higher Ti content, attributable to differing sputtering rates (Figure 5c). Given its lower atomic mass, Ti exhibits a higher sputtering yield, leading to preferential Ti deposition on the substrate. Compared with S‐NTO, point scans of the C‐NTO film reveal a higher Nb content, correlating with reduced transmittance (Figure 5d). Conversely, the higher Ti content in S‐NTO accounts for its superior optical transparency. Co‐sputtering enables fine‐tuned control over the relative distribution of Nb and Ti by adjusting the individual sputtering conditions of each target, leading to more uniform Nb incorporation. In the case of the G‐NTO sample, the elevated Ti content detected may result from the use of a 15 kV accelerating voltage during EDS, which penetrated deeper into the film, reaching the Ti‐rich regions located in the lower portion of the compositional gradient.

Figure 5e shows the absorption spectra of the three ETL films, with corresponding Tauc's plots derived to estimate their optical bandgaps. The G‐NTO film exhibits a slightly narrower bandgap (3.42 eV) compared with the S‐NTO (3.51 eV) and C‐NTO (3.58 eV) films, although all values remain within a comparable range (Figure 5f). This red‐shifted absorption edge in the G‐NTO film reflects a reduced bandgap, indicative of improved photon harvesting capability. Despite this reduction, the bandgap remains well above the main absorption range of the perovskite layer (e.g., >3.2 eV), ensuring that its absorption remains primarily within the UV spectrum and does not impede visible‐light absorption (500–800 nm).^[^ ^9^ ^]^ Consequently, G‐NTO can capture photons at longer wavelengths, approaching the visible spectrum. This enhances overall solar photon utilization and contributes to improved device performance. UPS measurements (Figure 5g,h) reveal significant variations in energy band alignment among the three ETL types. Based on the estimated bandgaps, the conduction band minimum (CBM) values were calculated. As shown in Figure 5i. This improved band alignment facilitates more efficient electron extraction. Furthermore, the Nb‐rich top layer of the G‐NTO contributes to enhanced ambient stability of the device. In addition to energy level matching, the internal charge transport properties of the ETL are critical for efficient extraction and injection of photogenerated electrons into the electrode, influencing the overall performance of the photovoltaic device.^[^ ^43^ ^]^ The electron mobility of the perovskite absorber (typically on the order of 10^3^ to 10^2^ cm^2^·V^−1^·s^−1^) significantly exceeds that of conventional TiO_2_‐based ETL.^[^ ^47^ ^]^ Incorporating Nb dopants into the TiO_2_ matrix has been shown to significantly enhance electron mobility. This is attributed to the partial substitution of Ti^4+^ with Nb^5+^, which introduces additional free electrons, increasing the carrier concentration and material conductivity.^[^ ^34^ ^]^ In addition, Nb incorporation may influence the concentration of *V*_O_, unctioning analogously to magnesium doping in tin oxide systems, as reported by Lan *et al.* ^[^ ^9^ ^]^ Higher electron mobility in the ETL accelerates charge extraction at the perovskite/ETL interface, improving overall charge dynamics. Accordingly, the upward shift of the CBM in G‐NTO enhances electron extraction and transport at the G‐NTO/perovskite interface, increasing the *V*_OC_ of the corresponding solar cell.^[^ ^48^ ^]^ These factors also promote improved electron injection from the perovskite into the ETL and reduce charge accumulation at the ETL surface. Regardless of the deposition method, the VBM remained relatively unchanged (Figure 5h), with all samples exhibiting VBM values of ≈3.62 eV. This consistency suggests that the valence band structures, predominantly governed by the amorphous phase, are generally similar among the three film types. The lower work function observed for NG‐NTO is more favorable for forming an effective interfacial contact with the perovskite layer, enhancing electron extraction efficiency. By integrating the bandgap estimations and UPS results, a comprehensive energy level diagram of the PSC device was constructed (Figure 5i). For G‐NTO, the Fermi level (EF) and VBM were elevated by 0.92 eV, while the CBM increased by 0.76 eV. Figure S10 (Supporting Information) compares the band structures of S‐NTO, C‐NTO, and G‐NTO, including their respective bandgaps and energy levels of CBM and VBM. Importantly, as the EF of the ETL in an n–i–p architecture rises, the energy offset between the ETL and HTL increases, strengthening the built‐in electric field. This enhancement facilitates more efficient charge separation at the junction, crucial for suppressing recombination losses and achieving high PCEs.^[^ ^45^ ^]^ Meanwhile, to further investigate the extent of band alignment modulation induced by the G‐NTO gradient structure, Figure S11 (Supporting Information) presents UPS measurements of the film deposited at Nb_2_O_5_ RF power of 50 W, representing the bottommost layer of the G‐NTO film. Based on these measurements, the conduction band position was determined. To visually illustrate the energy modulation range of G‐NTO, a schematic diagram of the band alignment was constructed, as shown in Figure S12 (Supporting Information). The gradual decrease of the conduction band from 5.05 to 4.28 eV creates a continuous energy gradient that facilitates electron transport. This gradient reduces the energy barrier at the ETL/perovskite interface, minimizes electron accumulation and recombination, and promotes more efficient electron extraction. It is worth noting that the energy band modulation range of WG‐NTO and NG‐NTO is expected to be the same, with the main difference arising from the number of modulation steps within the intermediate layers of the film. In the NG‐NTO film, the band alignment is modulated more gradually, leading to a smoother transition of energy levels across the film. Compared with abrupt band changes, the smooth transition in the NG‐NTO structure enables electrons to flow “downhill” along the energy gradient, enhancing charge transport and ultimately contributing to higher device performance. Consequently, raising the ETL CBM reduces the energy barrier for electron transfer from the perovskite, yielding improved energy alignment and more favorable carrier dynamics.

Notably, the NTO films were not subjected to high‐temperature annealing, which may partly account for their relatively lower optical transmittance. Nevertheless, the presence of a gradient doping structure significantly improves energy level alignment and electron transfer from the ETL to the electrode. Moreover, the amorphous or low‐crystallinity character of these films suppresses the formation of grain boundary traps, reducing carrier recombination and enhancing device performance. Although a slight decrease in transparency is observed, the reduction in UV light absorption and improvement in carrier extraction efficiency can effectively compensate for optical losses, provided that sufficient visible light reaches the perovskite absorber. Thus, gradient sputtering emerges as a promising strategy for bandgap engineering in ETLs, particularly for applications targeting enhanced photovoltaic conversion efficiency.

**Figure 6:**
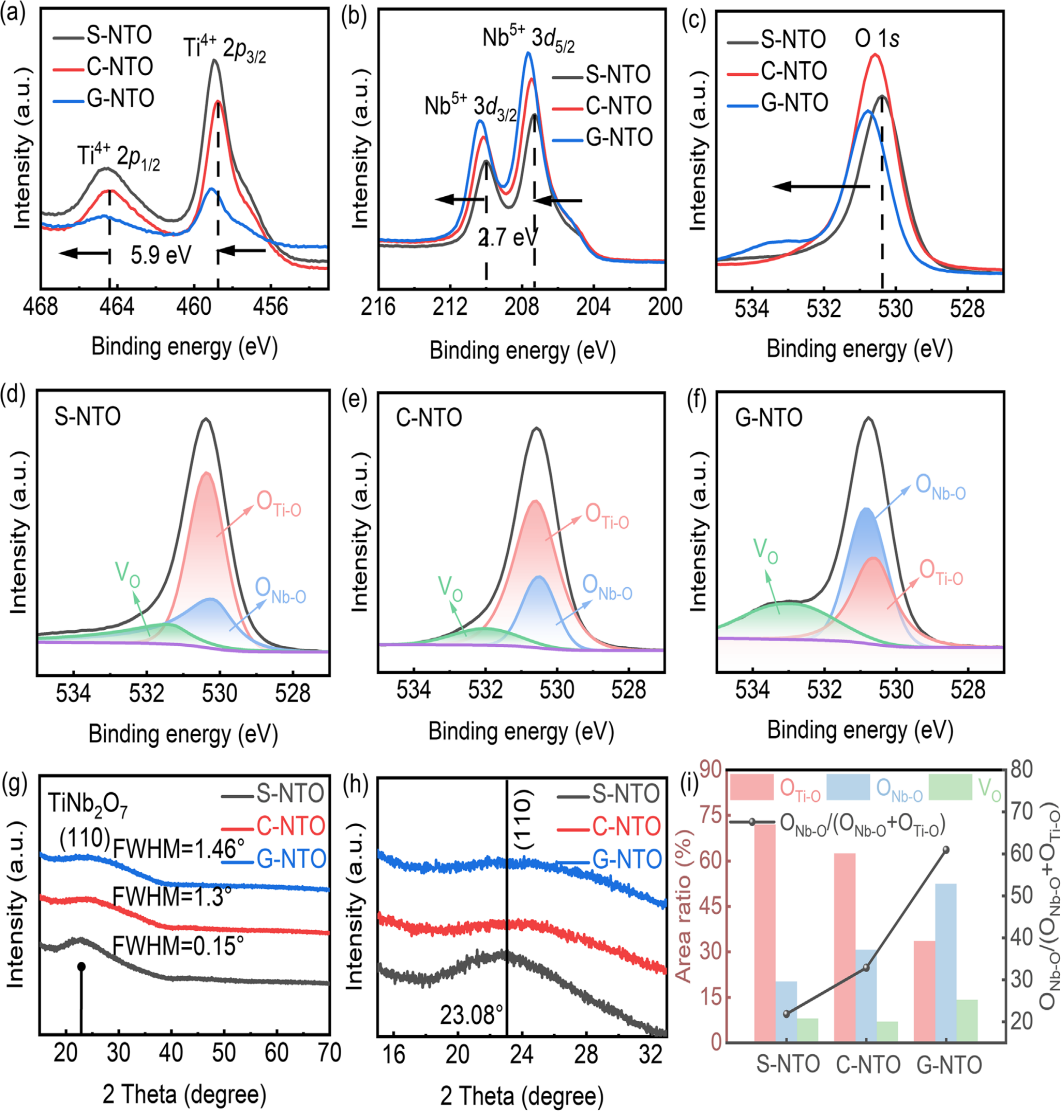
High‐resolution photoelectron spectroscopy (XPS) of a) Ti 2*p*, b) Nb 3*d*, and c) O 1*s* core levels of S‐NTO, C‐NTO, and G‐NTO ETLs. d–f) Fitting of the O 1*s* spectra of S‐NTO, C‐NTO, and G‐NTO ETLs. g,h) XRD results of S‐NTO, C‐NTO, and G‐NTO ETLs. i) Area ratios of *V*_O_, O_Nb‐O_, O_Ti‐O_, and the relative ratio of O_Nb‐O_/(O_Nb‐O_+O_Ti‐O_) calculated using the area of the fitted peaks of the O 1*s* orbits of S‐NTO, C‐NTO, and G‐NTO ETLs.

**Figure** **6**a–c show the high‐resolution X‐ray photoelectron spectroscopy (XPS) spectra of Nb‐doped TiO_2_ films (S‐NTO, C‐NTO, and G‐NTO), including the Ti 2*p*, Nb 3*d*, and O 1*s* core levels. A comparative analysis of the binding energies and peak intensities among the samples provides deeper insight into the correlation between chemical state, structural variation, and device performance. After Ar⁺ sputtering, a notable increase in overall XPS signal intensity is observed, particularly in the Ti 2*p* and Nb 3*d* regions (Figure S13, Supporting Information). This enhancement is primarily attributed to the removal of surface contaminants such as adventitious carbon and adsorbed species, which previously attenuated the XPS signals. The sputtering treatment exposes a more representative bulk‐like surface, improving signal fidelity and allowing for more accurate interpretation of the chemical states. Post‐cleaning, the binding energy scale of the spectra was recalibrated based on the C 1*s* peak position before sputtering (Figure S14, Supporting Information), using the standard reference value of 284.8 eV.^[^ ^9^ ^]^

In the Ti 2*p* photoelectron spectra, all three samples exhibit the characteristic doublet peaks of Ti^4+^ (Ti 2*p*_3/2_ ≈ 458.5 eV, Ti 2*p*_1/2_ ≈ 464.4 eV). Notably, in the G‐NTO sample, these peaks shift significantly toward higher binding energies (≈0.2–0.3 eV) and exhibit a significant reduction in intensity. Such a shift typically reflects a decrease in the electron cloud density surrounding Ti atoms, potentially arising from an increased concentration of *V*_O_ or enhanced structural disorder that alters the local coordination environment. Similarly, in the Nb 3*d* spectra, the characteristic Nb^5+^ peak in G‐NTO shifts slightly toward higher binding energy, accompanied by an increase in peak intensity. This indicates a higher Nb content and strengthened electronic interactions within the chemical environment. These trends are further corroborated by the O 1*s* spectra. As shown in the fitting results (Figure 6d–f), all three samples contain three primary components, attributed to lattice oxygen species (O_Ti‐O_ and O_Nb‐O_) and *V*_O_. Clearly, the relative proportions of the two elements agree well with the earlier EDS measurements. In the G‐NTO sample, a significant increase in the proportion of *V*_O_ is observed, alongside a decrease in lattice oxygen content, indicating a more pronounced oxygen deficiency. Moreover, the main O 1*s* peak in G‐NTO shifts toward higher binding energy, suggesting the presence of sub‐stoichiometric oxidation states and elevated *V*_O_ concentrations. Despite the fact that XPS analysis indicates G‐NTO possesses the highest degree of oxygen deficiency, the most structural disorder, and the largest density of defects, these seemingly unfavorable traits are counterbalanced by a precisely engineered compositional gradient and an amorphous matrix that collectively endow the material with exceptional optoelectronic properties. On one hand, the vertical gradient distribution of Ti and Nb enables finely tuned bandgap modulation. This facilitates directional electron transport within the ETL and optimizes band alignment with the perovskite absorber layer, effectively reducing interfacial energy barriers. On the other hand, a moderate *V*_O_ concentration can serve as shallow donor states, increasing carrier concentration. In addition, the amorphous structure minimizes the presence of grain boundary traps and recombination centers, enhancing electron mobility. Consequently, although the G‐NTO sample appears chemically incomplete based on surface analysis, its carefully tailored structural and compositional features yield superior interfacial charge extraction and transport capabilities, resulting in the highest photovoltaic conversion efficiency among the studied devices. This observation underscores the critical insight that performance optimization in functional oxide thin films should not be constrained solely by the pursuit of ideal stoichiometry. Rather, comprehensive strategies that holistically address band structure modulation, interfacial contact quality, and carrier dynamics must be emphasized. Crystallographic analyses further highlight these differences. The S‐NTO sample exhibits a pronounced diffraction peak near 23°, corresponding to the (110) plane of TiNb_2_O_7_ (Figure 6g,h), showing a strong preferential orientation and sharp peak profile, indicative of well‐defined crystallinity and a dominant crystalline phase.^[^ ^49^ ^]^ By contrast, the diffraction peak intensities of the C‐NTO and G‐NTO samples are significantly diminished, with broader peak widths, indicating reduced crystallinity. In particular, G‐NTO approaches an amorphous state. HRTEM (Figure 4i) reveals that the G‐NTO film consists primarily of an amorphous matrix interspersed with microcrystalline domains. The observed lattice fringe spacing of 0.37 nm corresponds to the (110) plane of TiNb_2_O_7_, confirming the presence of localized crystalline inclusions. While XPS data affirm the oxidation states of Ti^4+^ and Nb^5+^, consistent with TiNb_2_O_7_ stoichiometry, the XRD patterns show only broad, low‐intensity diffraction features. This further confirms a predominantly amorphous character with embedded microcrystallites. Among the three ETLs, G‐NTO exhibits the broadest (110) diffraction peak, with a full width at half maximum (FWHM) of 1.46°, indicating the lowest crystallinity and smallest grain size. By contrast, the smaller FWHM of S‐NTO is indicative of superior crystallinity, attributable to the “pre‐mixing followed by deposition” approach employed in single‐target sputtering, which favors crystal nucleation and growth. Conversely, co‐sputtering, characterized by simultaneous deposition and atomic rearrangement, is more susceptible to the formation of disordered structures, accounting for the reduced crystallinity observed in C‐NTO and G‐NTO. Notably, amorphous ETLs exhibit a smoother surface morphology, which enhances interfacial contact with the perovskite absorber layer. This morphological advantage facilitates efficient charge separation and carrier transport across the interface. In addition, the suppression of grain boundary formation in amorphous films reduces trap‐state densities, improving the *V*_OC_ and FF of the device.^[^ ^50^ ^]^ To further quantify chemical state evolution across the different samples, the area ratios of *V*_O_, O_Ti‐O_, and O_Nb‐O_ peaks were calculated, as shown in Figure 6i. A clear trend is observed: the proportion of O_Nb‐O_ progressively increases from S‐NTO to G‐NTO. This trend parallels the observed intensification and binding energy shifts in the Nb 3*d* spectra, confirming that Nb doping becomes more pronounced and more uniformly distributed in G‐NTO. Simultaneously, the chemical environment of Nb appears increasingly stabilized. Notably, this increase in O_Nb‐O_ content directly correlates with improved device performance. This offers compelling evidence that precise control over doping distribution and local atomic configuration can yield substantial improvements in charge transport pathways and energy level alignment—critical factors in maximizing overall device efficiency.

Collectively, the XRD and HRTEM results confirm that all NTO films exhibit relatively low crystallinity, with an amorphous matrix hosting only a small amount of TiNb_2_O_7_ nanocrystals. On this basis, we conducted a deeper investigation into how different film fabrication strategies influence the microstructural evolution of the films and, by extension, the photovoltaic performance of the resulting devices.

**Figure 7:**
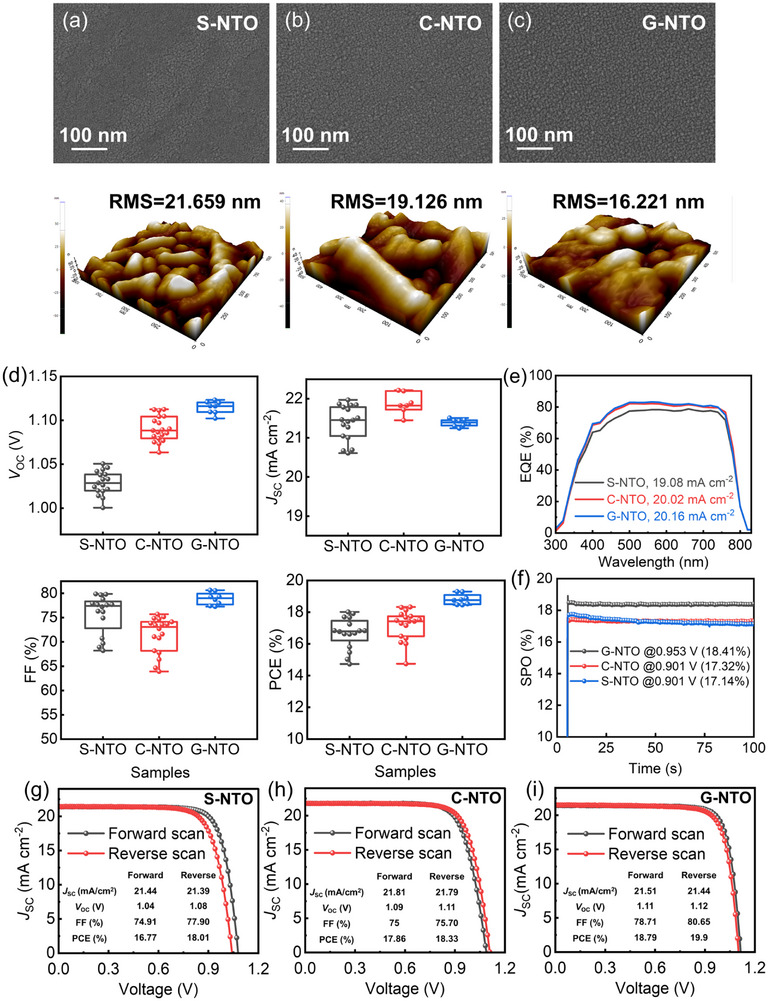
a–c) SEM images and corresponding atomic force microscopy (AFM) topographies (with root‐mean‐square (RMS) roughness values) of S‐NTO, C‐NTO, and G‐NTO thin films. d) Box plots of photovoltaic parameters for PSCs incorporating S‐NTO, C‐NTO, and G‐NTO as ETLs. e) external quantum efficiency (EQE) spectrum of the PSC device fabricated with S‐NTO, C‐NTO, and G‐NTO. f) Steady power output (SPO) tracking curves of PSCs with different NTO‐based ETLs under continuous illumination. g–i) *J–V* curves of the champion PSCs incorporating S‐NTO, C‐NTO, and G‐NTO ETLs.

**Figure** **7** summarizes the comparative characterization of the S‐NTO, C‐NTO, and G‐NTO ETLs. Figure 7a–c present the SEM images and atomic force microscopy (AFM) surface topographies of the three NTO films. The S‐NTO film exhibits a relatively coarse surface morphology, featuring a compact yet disordered microstructure, with a root‐mean‐square (RMS) roughness of 21.659 nm, as determined through AFM analysis. By contrast, the co‐sputtered C‐NTO film exhibits improved morphological uniformity and a reduced RMS roughness of 19.126 nm. Notably, the G‐NTO film, fabricated via a compositional gradient deposition strategy, exhibits the most compact and smooth surface morphology, with the lowest RMS roughness of 16.221 nm. These observations highlight that even within amorphous‐dominated systems, interface quality can be significantly improved through precise control of the deposition process. Figure 7d presents box plots of the photovoltaic parameters derived from PSCs incorporating the different NTO ETLs. Statistically, G‐NTO‐based devices deliver the most favorable performance. PSCs utilizing ETLs with more finely graded compositional profiles demonstrate higher FoM values (Figure S8, Supporting Information) and enhanced photovoltaic performance (Figure 4d) (n = 15 for each condition). The number of samples marked in the figure may seem inconsistent, but the actual number of samples produced is the same. The smaller number is because some individual cell test results were deleted as they were not satisfactory. Although their *J*_SC_ is marginally lower than that of C‐NTO devices, they exhibit a significantly enhanced FF, resulting in the highest PCE. This performance enhancement reflects superior charge extraction and transport characteristics. By contrast, C‐NTO‐based devices achieve a higher *J*_SC_, indicative of efficient photogenerated carrier collection. However, they suffer from lower FF and greater variability in device performance, which can be attributed to interfacial inconsistencies or residual defects. S‐NTO‐based devices consistently yield the lowest performance, underscoring the limitations imposed by suboptimal film morphology and interfacial quality. Figure 7e shows the external quantum efficiency (EQE) spectra for devices employing the different NTO ETLs. All devices demonstrate broad photoresponses spanning ≈350 to 800 nm, indicative of efficient light harvesting and consistent with an optical bandgap of ≈1.55 eV for the perovskite absorber.^[^ ^51^ ^]^ Among them, the G‐NTO device achieves the highest EQE across the entire spectral range, suggesting enhanced electron extraction efficiency and suppressed interfacial recombination, attributable to its compositional gradient architecture. Conversely, devices based on C‐NTO and S‐NTO exhibit slightly diminished EQE responses, with S‐NTO showing the weakest performance, particularly in the short‐wavelength region. The integrated *J*_SC_ values derived from EQE measurements follow the same trend, reinforcing the efficacy of gradient doping in facilitating improved carrier collection. Further evidence of this trend is presented in Figure S15 (Supporting Information). which illustrates maximum power point (MPP) tracking results. Devices employing G‐NTO ETLs consistently maintain higher and more stable output under operational conditions. The steady‐state power output (SPO) curves in Figure 7f corroborate this observation, revealing that G‐NTO‐based devices sustain higher output power densities, reflecting enhanced light responsivity and interfacial charge transport efficiency. Unencapsulated devices were tracked at 25 ± 5 °C and 30 ± 5% RH under MPP conditions. While overall stability was limited, Figure S16 (Supporting Information) shows G‐NTO effectively suppresses PCE decay, outperforming uniform or conventional doping likely by reducing interfacial defects, optimizing band alignment, and facilitating electron transport. ETL films were further tested under accelerated aging (85 °C, 85% RH) (Figure S16, Supporting Information). UV–vis and Hall measurements on FTO/glass revealed that S‐NTO and C‐NTO suffered optical degradation and mobility loss due to trap accumulation, whereas G‐NTO maintained stable absorption, transmittance, and carrier mobility. Normalized resistivity and sheet resistance remained constant, indicating that gradient doping suppresses defect formation and stabilizes electron transport pathways. Figure 7g–i present the J–V curves of the best‐performing PSCs incorporating each ETL variant. The G‐NTO device achieves the highest PCE, primarily attributed to its higher *V*_OC_ and FF, despite a slightly lower *J*_SC_ compared with C‐NTO. The C‐NTO device achieves the highest *J*_SC_, confirming efficient photocharge generation and collection. However, its relatively low FF suggests increased recombination losses or interfacial impedance. The S‐NTO device exhibits the weakest overall performance, attributable to its higher surface roughness and fewer continuous charge transport pathways, which hinder efficient carrier extraction. Table S3 (Supporting Information) summarizes reported PCEs for various doped metal oxide ETLs, showing that the G‐NTO film prepared here by magnetron sputtering achieves an efficiency of 19.90%, comparable to other similar ETL‐modified PSCs and clearly demonstrating the effectiveness of our approach. In summary, although all NTO films in this study predominantly exhibit amorphous character, it is evident that implementing advanced deposition strategies—most notably, compositional gradient engineering—can significantly improve surface morphology, charge transport pathways, and overall device performance. These findings underscore the crucial role of microstructural control in ETL design and introduce a compelling paradigm for harnessing amorphous oxide materials in developing high‐efficiency, stable PSCs.

## 3 Conclusion

This study systematically compares three different fabrication methodologies for NTO ETLs in PSCs, including single‐target sputtering, conventional co‐sputtering, and dynamically controlled gradient co‐sputtering. The findings reveal that through real‐time control of sputtering power during deposition, a compositional gradient NTO film was successfully engineered, featuring a Ti‐rich bottom layer and a Nb‐rich top layer, without necessitating high‐temperature post‐annealing. This engineered band gradient significantly improves interfacial energy level alignment and facilitates more efficient electron transport pathways. Consequently, the gradient architecture promotes improved charge separation and interfacial transfer, enhancing device performance. Compared with PSCs employing single‐target and conventional co‐sputtered ETLs, devices incorporating the gradient NTO ETL exhibited notable enhancements in *V*_OC_ and FF, with PCE improvements of ≈7.4% and 6.3%, respectively. A peak PCE of 19.9% and a *V*_OC_ of 1.12 V were achieved. Despite all NTO films exhibiting predominantly amorphous microstructures, the gradient doping strategy effectively mitigated the intrinsic limitations of amorphous materials, including their relatively low carrier mobility, by enhancing charge carrier transport, suppressing interfacial recombination, and reducing series resistance. These results underscore a robust structure–performance relationship enabled by compositional modulation. Furthermore, the proposed gradient doping methodology streamlines the fabrication process and demonstrates excellent compatibility with low‐temperature and flexible device applications. This work offers valuable insights into the rational design of functional metal oxide thin films and presents a promising route for low thermal‐budget processing strategies in next‐generation optoelectronic devices.

## 4 Experimental Section

### Materials

Tin (IV) oxide (SnO_2_, 15% in H_2_O colloidal dispersion, Alfa Aesar), methylammonium chloride (MACl,99.99%, GreatCell Solar), formamidinium iodide (FAI; 99.99%, GreatCell Solar), lead(II) iodide (PbI_2_; 99.99%, TCI), n‐octylammonium bromide (OABr, 99%, GreatCell Solar), silver (Ag, 99.99%), N, N‐dimethylformamide (DMF, anhydrous, 99.8%, Sigma–Aldrich), dimethyl sulfoxide (DMSO, anhydrous, 99.9%, Sigma–Aldrich), spiro‐OMeTAD (2,29,7,79‐tetrakis (N, N‐di‐p‐methoxyphenylamine)‐9,9‐spirobifluorene, 99.5%, Derthon), 4‐tert‐butylpyridine (4‐tBP, 98.0%), and lithium bis (trifluoromethane sulfonyl) imide (Li‐TFSi, 99.95%, Sigma–Aldrich).

### RF Magnetron Sputtering of the NTO Films

The NTO thin films with varied Nb concentrations were prepared on FTO coated glass substrates using conventional radio frequency (RF) magnetron sputtering at room temperature (25 °C). The FTO coated glass substrates (AMG Korea) were cleaned with acetone, isopropyl alcohol, and methanol in an ultrasonic cleaner for 10 min and dried in ambient N_2_ gas (99.99% purity). After the cleaned FTO coated glass substrates were loaded into a 4 in. load lock chamber connected to the main magnetron sputtering chamber (Nnsvacuum), the base pressure was pumped down to ≈1.5 × 10^−6^ Torr. Prior to deposition, 4 in. TiO_2_ and Nb_2_O_5_ targets (Dasom, 99.99% purity) were pre‐sputtered in a pure Ar atmosphere for 10 min to remove contaminants on their surfaces. Ar gas (99.999% purity) and O_2_ gas (99.999% purity) were used as sputtering gases. Ar gas and O_2_ gas were introduced via two separate nozzles so that the partial pressure could be adjusted. Single‐target sputtering employed a NbTiO_x_ target (Nb 43.7 wt.%‐doped TiO_2_) with a base pressure of 1.5 × 10^−6^ Torr and deposition parameters of 100, 150, 200 W RF power, 2, 3, 4 mTorr working pressure, and 0, 0.2, 0.4 sccm O_2_ partial pressure to deposit NTO films on FTO coated glass substrates. Co‐sputtering used a TiO_2_ and Nb_2_O_5_ dual‐target system, first optimizing TiO_2_ deposition parameters (base pressure 1.5 × 10^−6^ Torr; RF power of 100, 150, 200 W; Working pressure of 2, 3, 4 mTorr; O_2_ partial pressure of 0, 0.2, 0.4 sccm) on FTO coated glass substrates, then adjusting Nb_2_O_5_ sputtering power of 50, 75, 100, 125, and 150 W. Building upon the co‐sputtering process, we designed two types of graded NTO films with different transition zones by progressively varying the sputtering powers of the dual targets (TiO_2_‐Nb_2_O_5_) from bottom to top as follows: (1) 200‐50 W → 175‐75 W → 150‐100 W → 100‐150 W; (2) 200‐50 W → 150‐100 W → 100‐150 W. To better distinguish the films prepared using the three deposition methods, the samples fabricated under each method were designated as single‐target sputtered (S‐NTO), co‐sputtered (C‐NTO), and gradient co‐sputtered (G‐NTO), respectively.

### Analysis of the NTO Films

The thickness was measured using an Alpha‐step 250 profilometer. The electrical properties were evaluated with an HMS‐4500 Hall measurement system, and optical property analysis was performed by a UV‐670 UV–vis spectrophotometer. The surface morphology was observed using a JSM‐7600F field emission scanning electron microscope (FESEM) at a 15 kV accelerating voltage (samples coated with 30 s Pt deposition). Elemental composition analysis was conducted via integrated energy dispersive X‐ray spectroscopy (EDS). Chemical state determination was performed via K‐alpha X‐ray photoelectron spectroscopy (XPS). The crystal structures were characterized using a D8 Advance grazing‐incidence X‐ray diffraction (GI‐XRD), and the work function was measured via ultraviolet photoelectron spectroscopy (UPS) with a He I source (21.2 eV). High‐resolution transmission electron microscopy (HRTEM, JEM‐2100F, JEOL) was employed to analyze the cross‐sectional structure of the NTO films. The samples were prepared using focused ion beam (FIB, FEI Nova Nanolab 600) on an FEI Quanta 3D system, following Pt coating for surface protection. HRTEM imaging, selected area electron diffraction (SAED), and energy‐dispersive X‐ray spectroscopy (EDX) mapping were conducted at an accelerating voltage of 200 kV to investigate the elemental distribution of the films. In addition, surface morphology and roughness were examined using atomic force microscopy (AFM, NX‐10). The optoelectronic stability of the ETL was evaluated under 85 °C and 85% RH testing conditions climatic chamber (TH‐PE‐025, JEIO TECH).

### Preparation of Perovskite Precursor Solution

The 1.5 M perovskite precursor FAPbI_3_ solution was prepared by dissolving the corresponding amounts of FAI (258 mg) and PbI_2_ (691.5 mg) in the DMF/DMSO (800 µL/200 µL), and 30% MACl (30.4 mg) was added as the additive. The solution was filtered using a syringe filter with a 0.2 µm pore size (Hyundai Micro).

### Fabrication of Normal Perovskite Solar Cells (n‐i‐p devices)

The SnO_2_ colloidal solution was diluted with deionized water at a 1:3 volume ratio. A 1 mL portion of the SnO_2_ solution was dispensed onto the cleaned ITO glass and spin‐coated at 3000 rpm for 20 s, followed by annealing at 150 °C for 30 min. The annealed SnO_2_ film was treated with oxygen plasma for 10 min before the coating of the perovskite layer. The prepared perovskite solution (75 µL) was spin‐coated onto the substrates at 5000 rpm for 50 s. Ten seconds after starting the spin‐coating process, 800 µL of diethyl ether was applied. The film was annealed using a two‐step process. The first annealing step was performed in a glove box at 60 °C for 3 min immediately after spin‐coating. The devices were then transferred to a dry room for the second annealing step at 150 °C for 10 min. The dry room conditions were strictly maintained at 20 °C and 15% relative humidity. After the perovskite deposition, the HTM was deposited by spin‐coating at 3000 rpm for 30 s. The HTM solution consists of 43.4 mg spiro‐OMeTAD, 17 µL 4‐tBP, and 10 µL of 540 mg mL^−1^ Li‐TFSi acetonitrile solution dissolved in 500 mL chlorobenzene. Finally, Ag (50 nm) was thermally evaporated on the top of the device to form the back contact.

### Device Characterization

The photocurrent density‐voltage (*J–V*) measurements were conducted under AM 1.5G 1 sun (100 mW cm^−2^) illumination, using a class AAA solar simulator (Oriel Sol 3A) equipped with a 450 W xenon lamp (Newport 6280NS) and a Keithley 2400 source meter. *J–V* curves for the PSCs were recorded at a scan rate of 0.1 V/s, with the voltage sweep ranging from −0.1 to 1.2 V (forward scan) and from 1.2 to −0.1 V (reverse scan). Light intensity calibration was performed using an NREL‐calibrated silicon reference solar cell. No preconditioning steps, such as applied bias voltage or light soaking, were conducted before measurements. The PSCs were tested with a metal mask (aperture area of 0.125 cm^2^) in ambient conditions (temperature≅25 °C, relative humidity≅30%). The operational stability of the PSCs was recorded at maximum power point tracking (MPPT) using a 100 mW cm^−2^ white LED lamp (Wuhan 91 PVKSolar Technology Co., Ltd).

## Conflict of Interest

The authors declare no conflict of interest.

## Author Contributions

H.‐K.K. conceived and directed the project. H.‐K.K. and J.W.L supervised the study. F.L. fabricated and characterized all NTO films. D.Z. fabricated the perovskite devices and measured their performance. S.C. aided in the device fabrication and material characterization. F.L. performed the Hall, UV–vis, AFM, FE‐SEM, XRD, XPS, UPS, and HRTEM measurements. Y.L., J.‐Y.K., J.J., H.‐J.J., S.L., J.‐Y.C. contributed to the investigation. F.L. analyzed the data and wrote the manuscript. All the authors have read and commented on this manuscript.

## Supporting information



Supporting Information

## Data Availability

The data that support the findings of this study are available from the corresponding author upon reasonable request.
