# Supplementary material for: Annealing‐Free Gradient Doping Strategy to Build Amorphous Nb:TiOX Electron Transport Layer for Efficient Perovskite Solar Cells
Source: Small. 2025 Nov 6;21(51):e10319. doi: 10.1002/smll.202510319 (PMC12723345; doi:10.1002/smll.202510319)
Supplement: Supplementary file 1 — Supporting Information [file SMLL-21-e10319-s001.docx]

Supporting Information

*Fang Luo^1^, Dongbo Zhang^2^, Shaozheng Chen^1^, Yewon Lee^1^, Jun-Hyeok Kang^1^, Joon Jang^1^, Hyun-Jung Jung^1^, Seungmin Lee^1^, Ji-Yoon Chae^1^, Jin-Wook Lee^2^ and Han-Ki Kim^1, 3, *^*

^1^School of Advanced Materials Science and Engineering, Sungkyunkwan University, Suwon, Gyunggi-do 16419, Republic of Korea
^2^SKKU Advanced Institute of Nanotechnology, Sungkyunkwan University, Suwon, Gyunggi-do 16419, Republic of Korea
^3^Department of Display Engineering, Sungkyunkwan University, Suwon, Gyunggi-do 16419, Republic of Korea


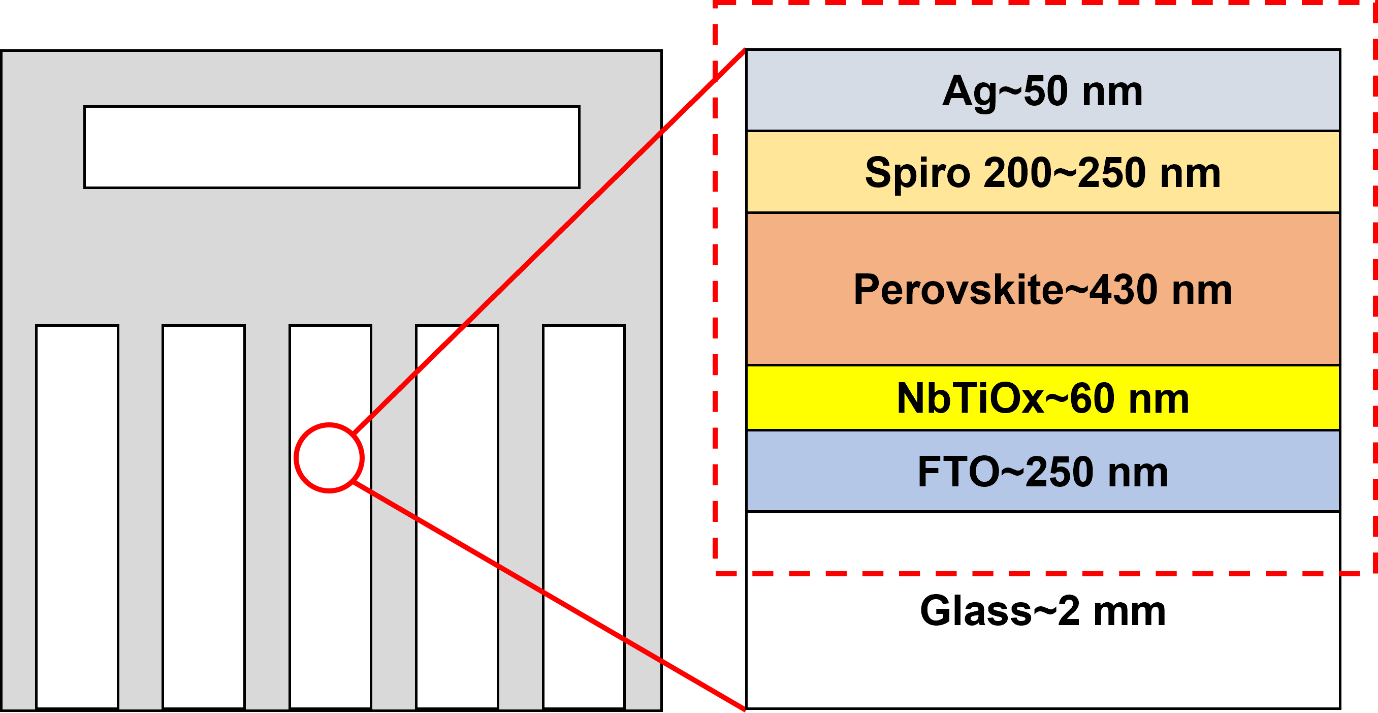


Fig. S1. Schematic of the PSC structure with NbTiOx buffer layer.


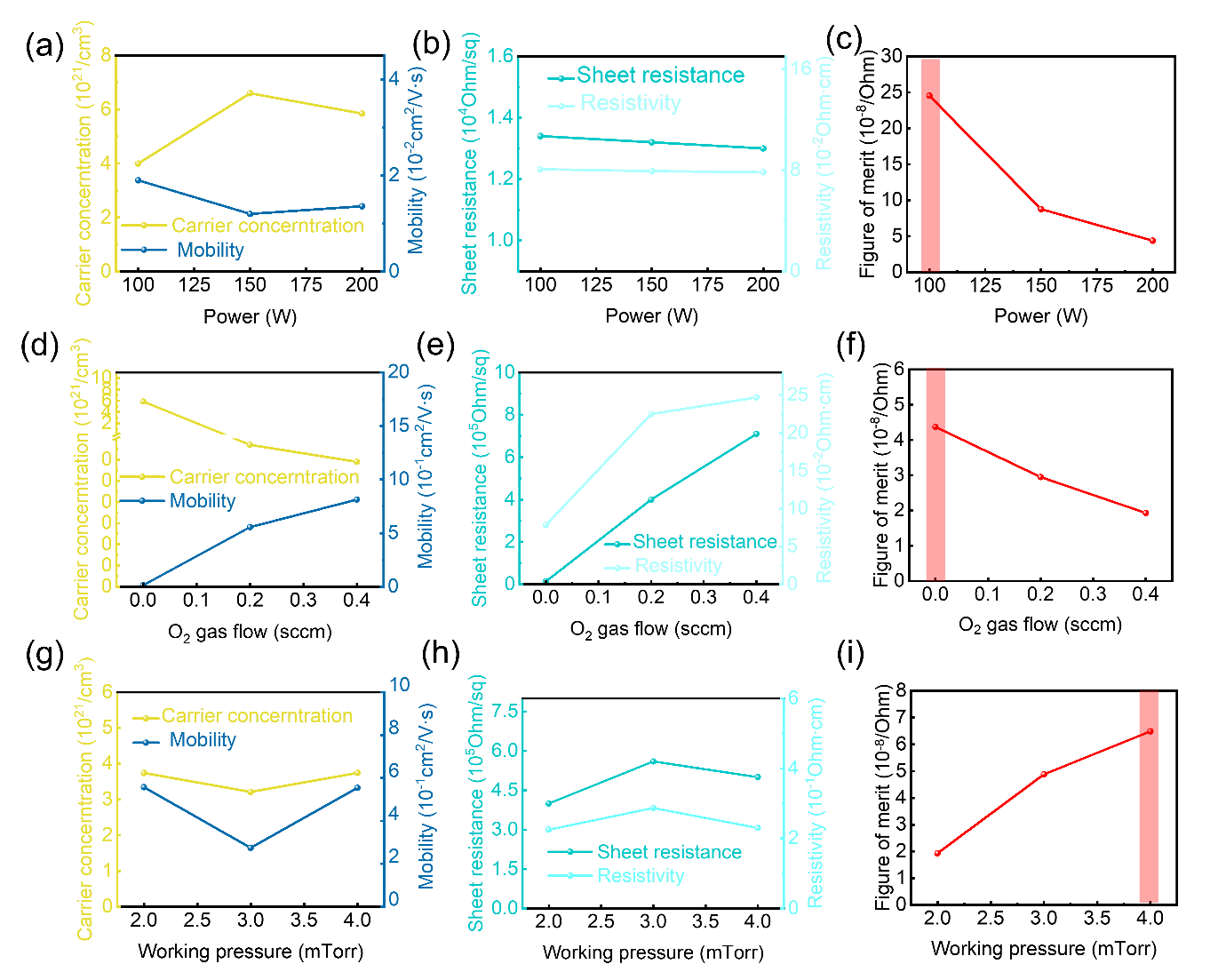


Fig. S2. Hall measurement results and FoM values of S-NTO films under different sputtering parameters. (a–c) Different RF powers. (d–f) Different O_2_ gas flow. (g–i) Different working pressures.


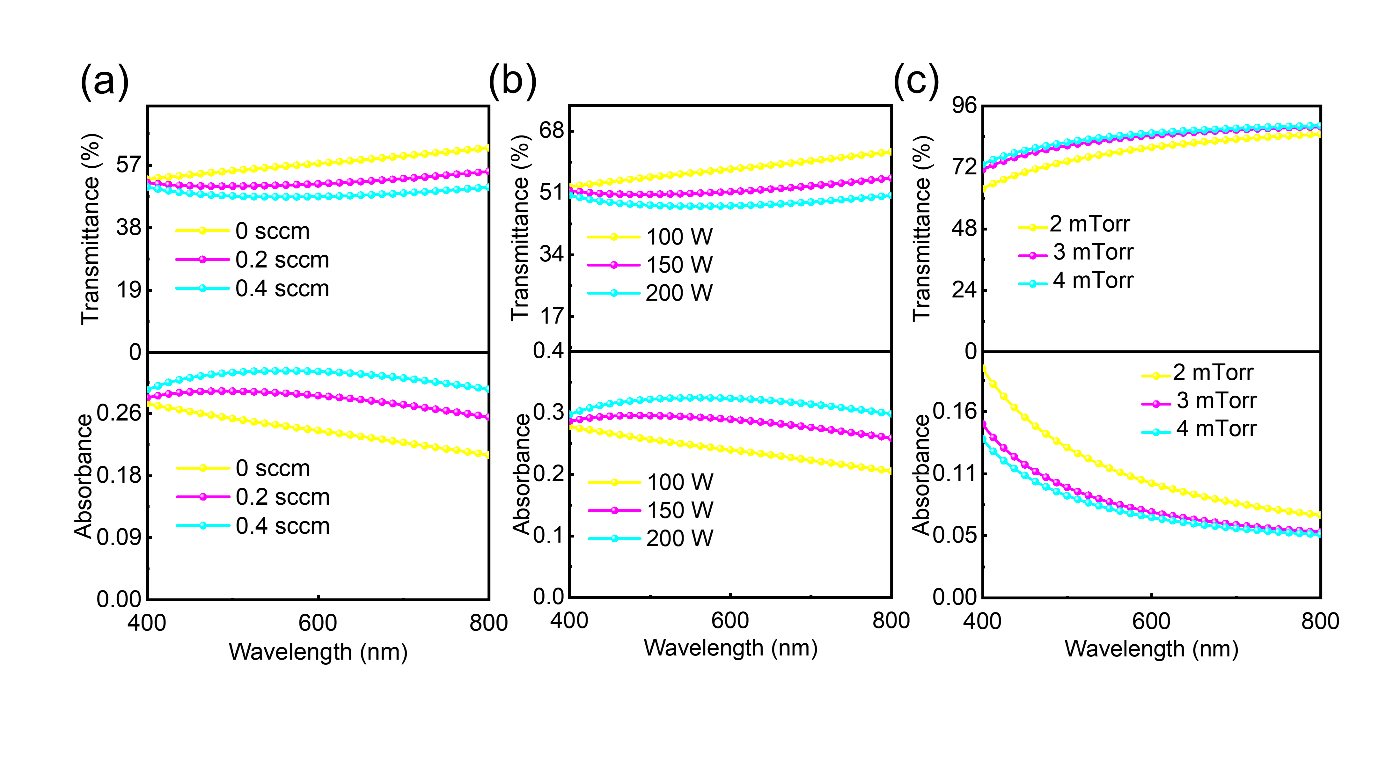


Fig. S3. Optical properties of S-NTO films under different sputtering parameters. (a) Different RF powers. (b) Different O_2_ gas flows. (c) Different working pressures.


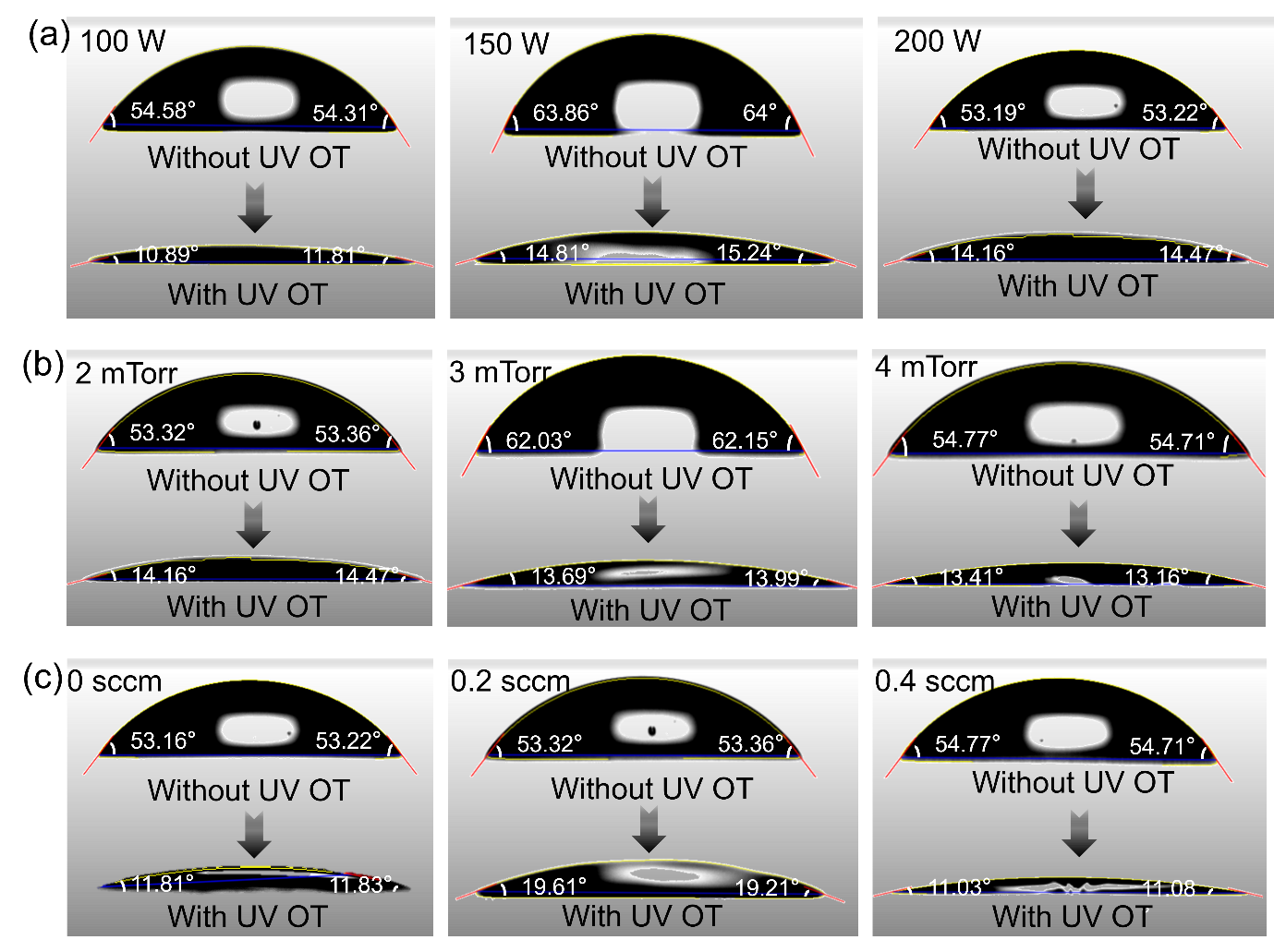


Fig. S4. Water contact angles of S-NTO films under different sputtering parameters. (a) Different RF powers. (b) Different working pressures. (c) Different O_2_ gas flows.


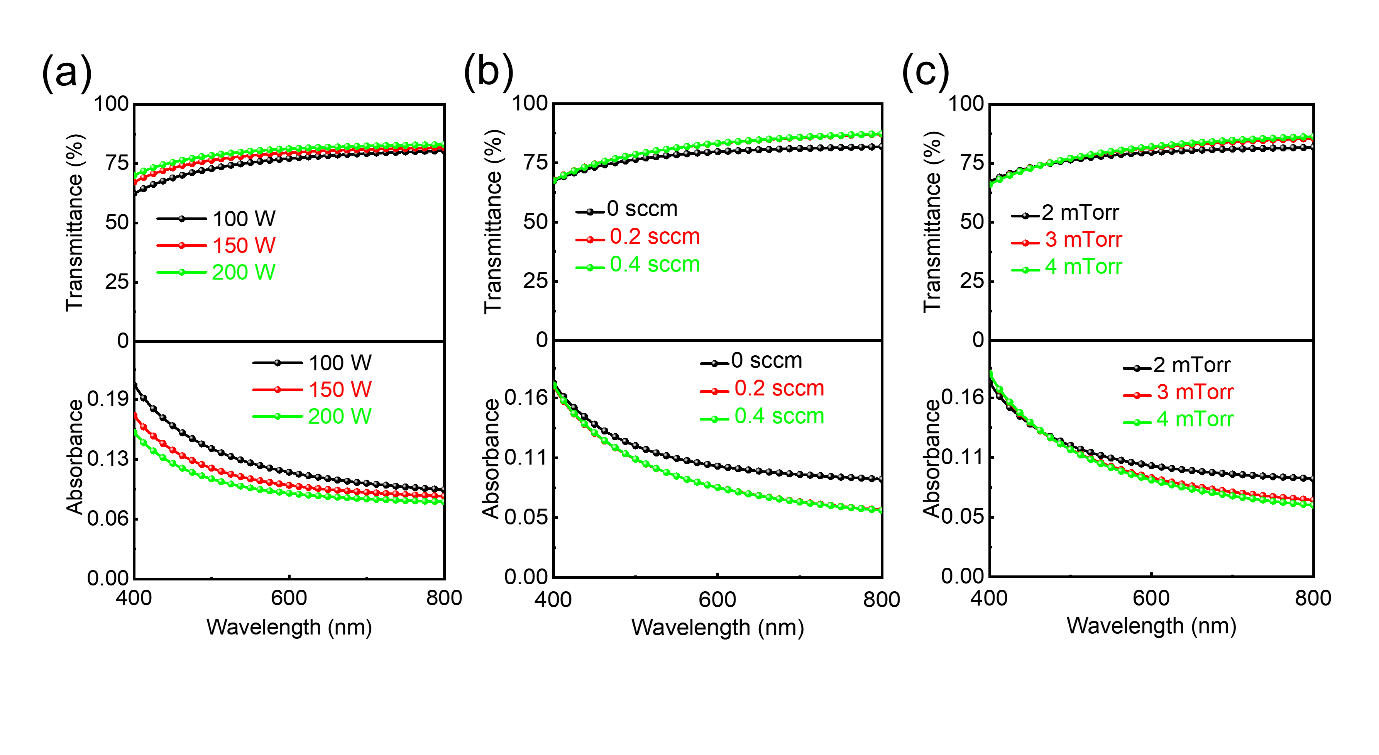


Fig. S5. Optical properties of TiO_2_ films under different sputtering parameters. (a) Different RF powers. (b) Different O_2_ gas flows. (c) Different working pressures.


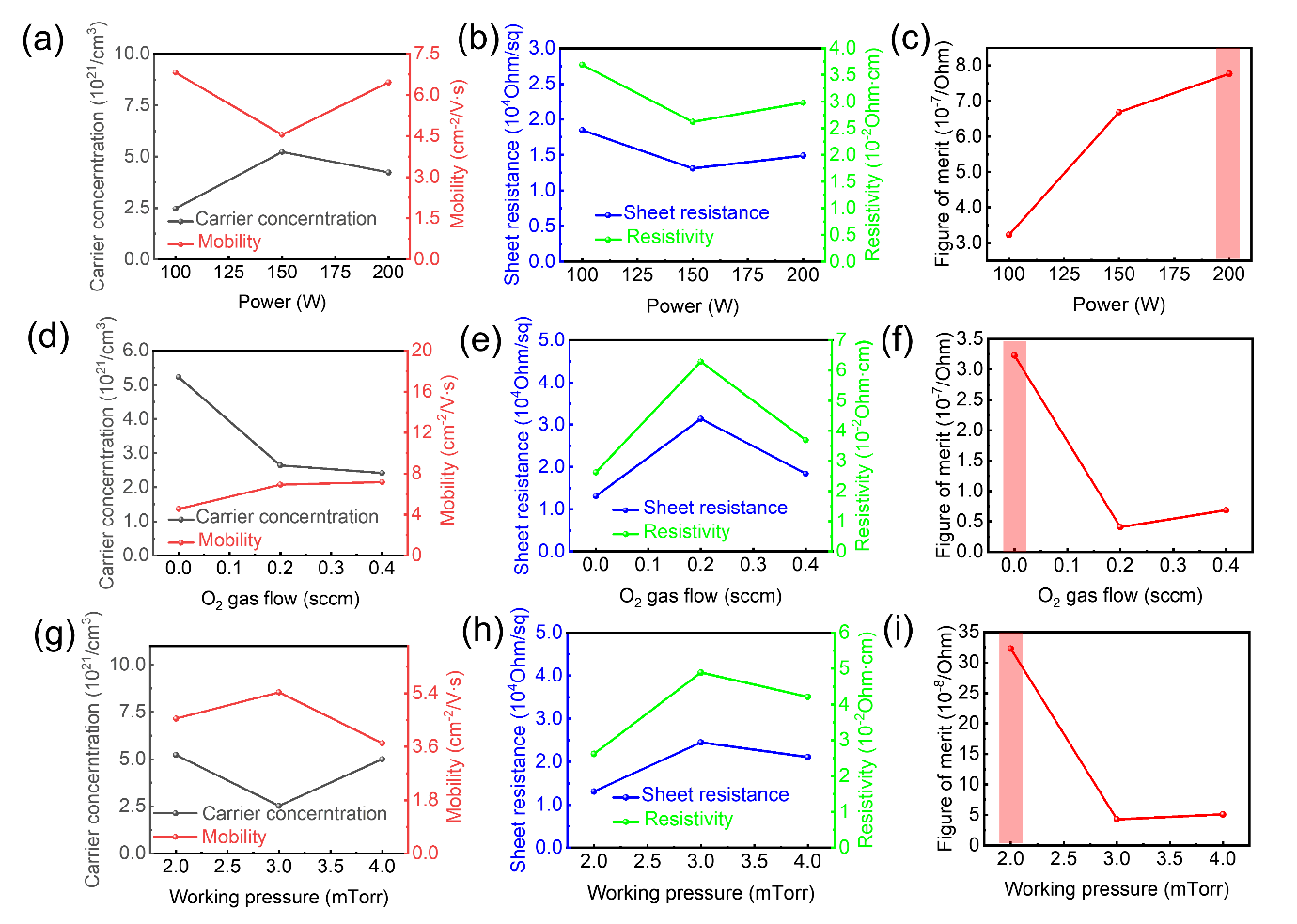


Fig. S6. Hall measurement results and FoM values of TiO_2_ films under different sputtering parameters. (a–c) Different RF powers. (d–f) Different O_2_ gas flows. (g–i) Different working pressures.


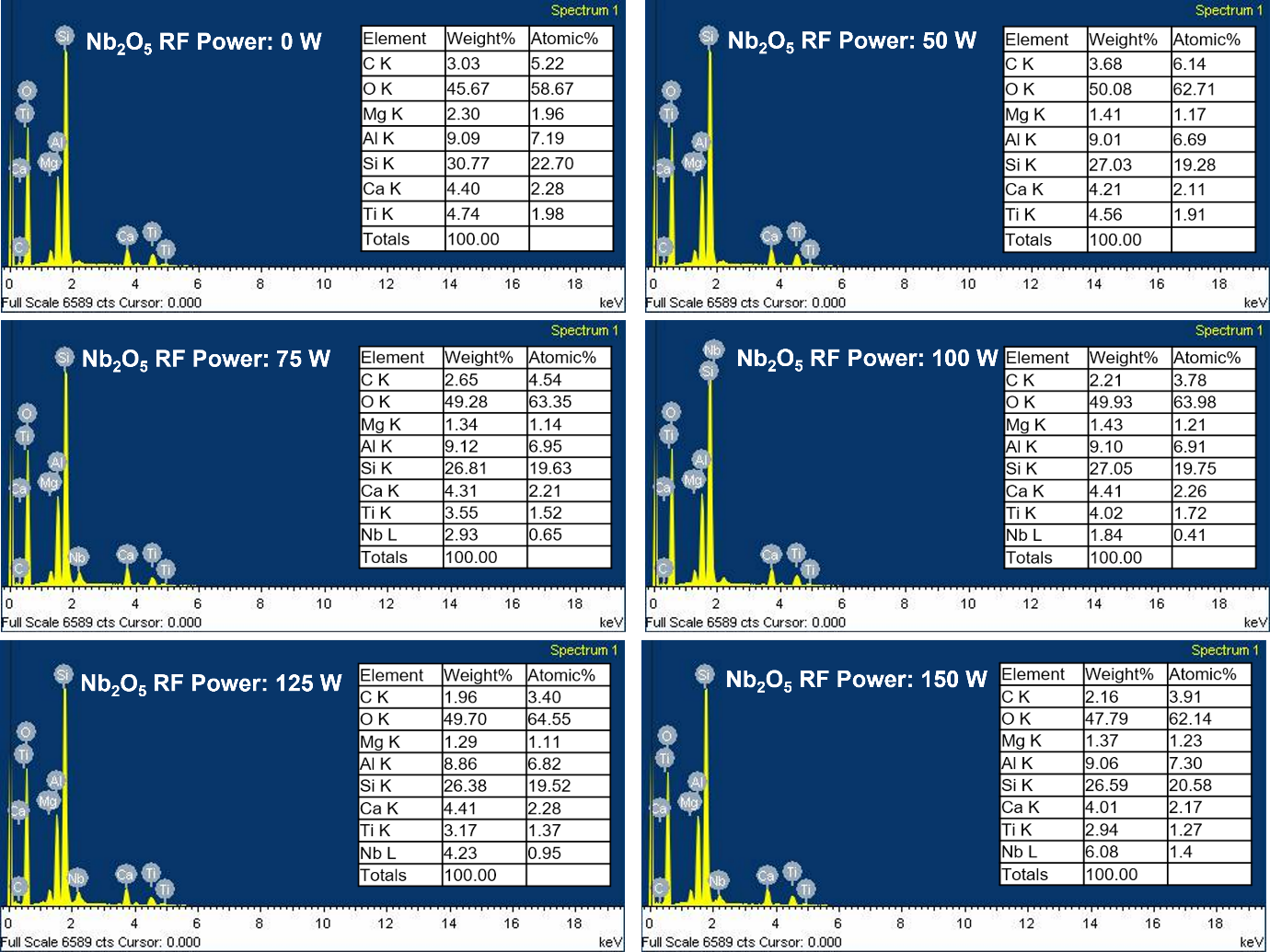


Fig. S7. EDS analysis results of C-NTO films according to the RF power applied to the Nb_2_O_5_ target.


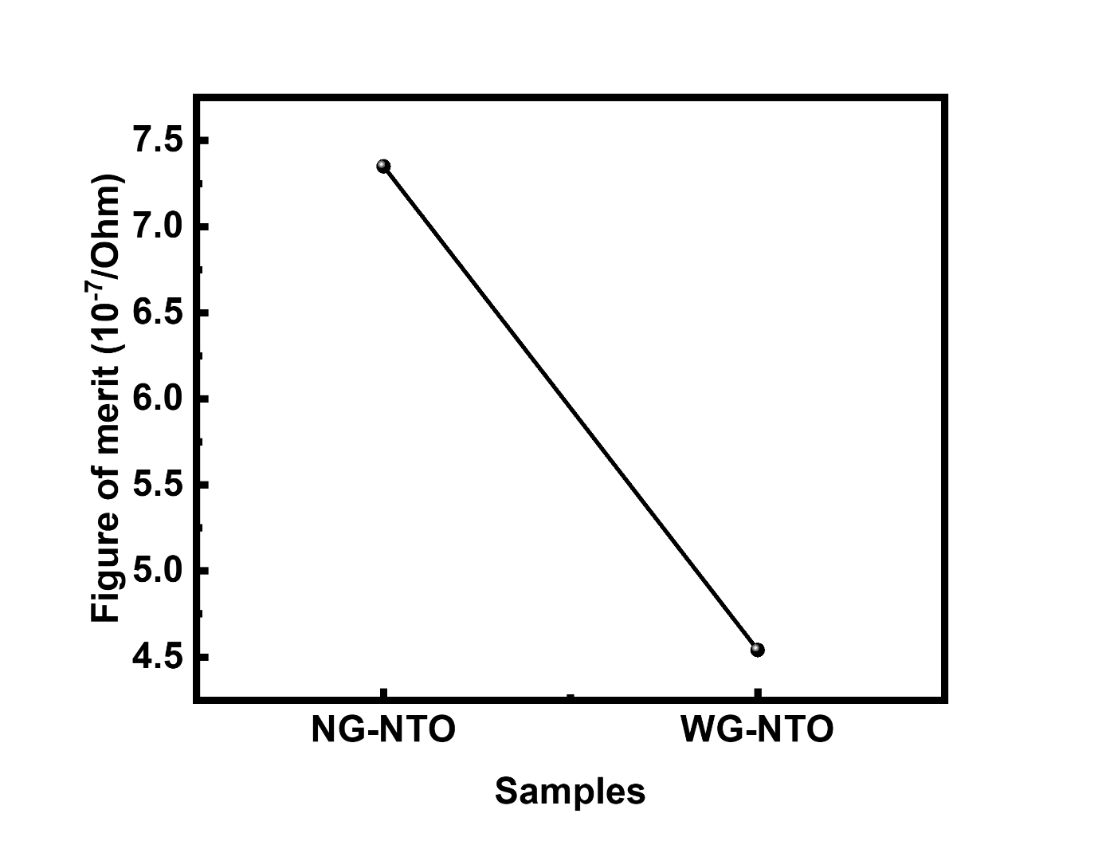


Fig. S8. FoM values of NG-NTO and WG-NTO films.


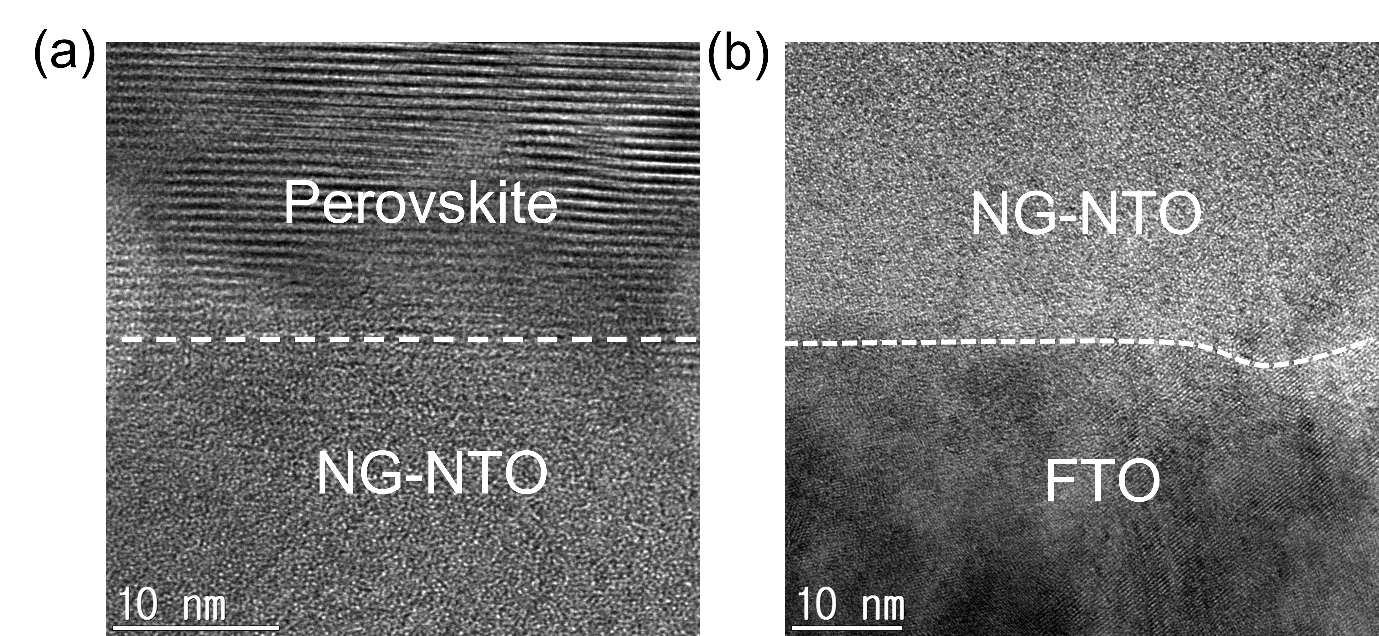


Fig. S9. HRTEM images. (a) Interface between perovskite and NG-NTO. (b) Interfaces between NG-NTO and FTO.


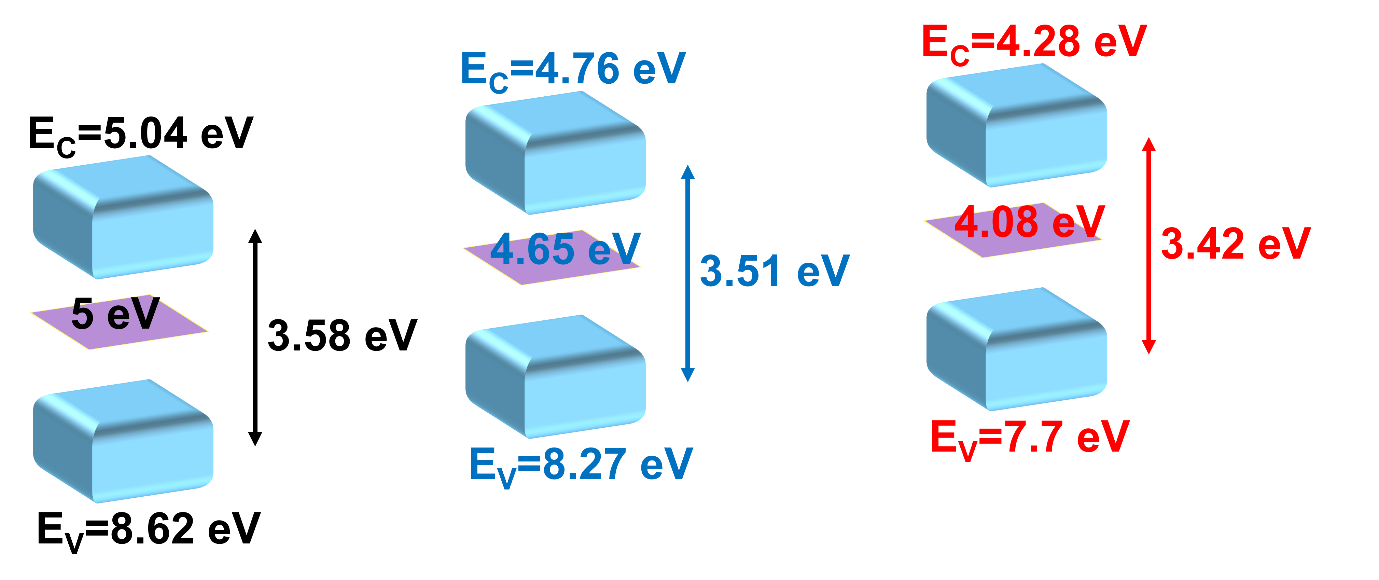


Fig. S10. Energy level alignment of S-NTO, C-NTO and G-NTO films.


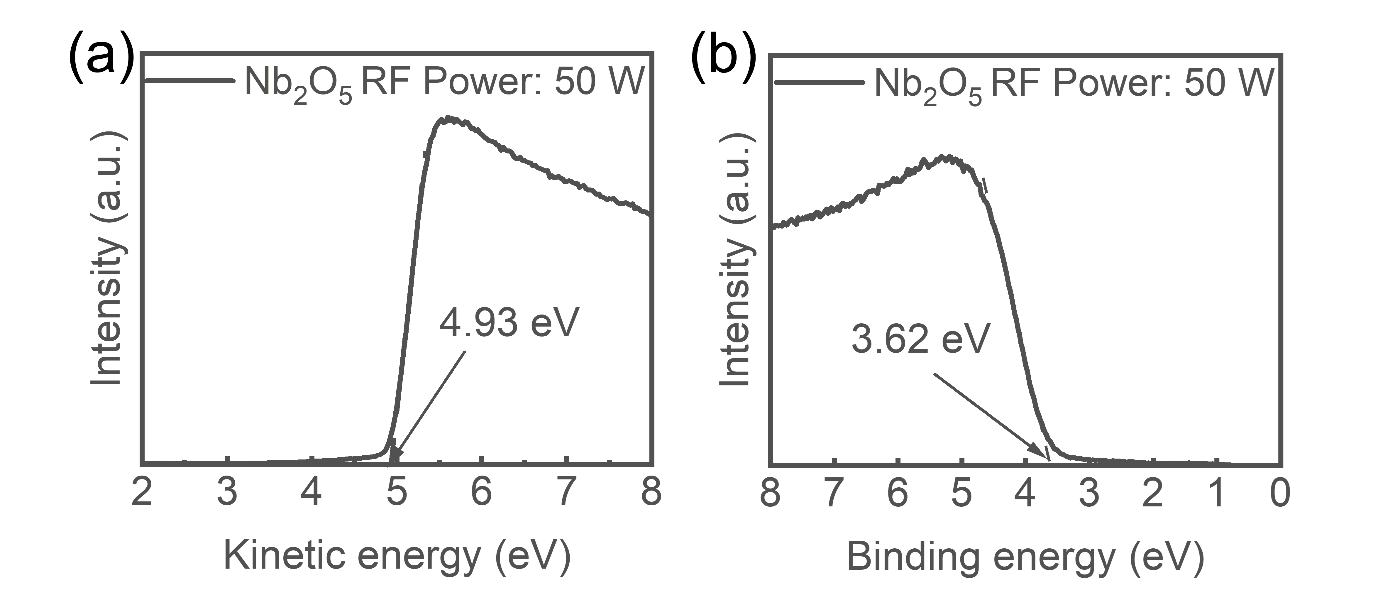


Fig. S11. (a) Secondary-electron cut-off region and (b) valence band region.


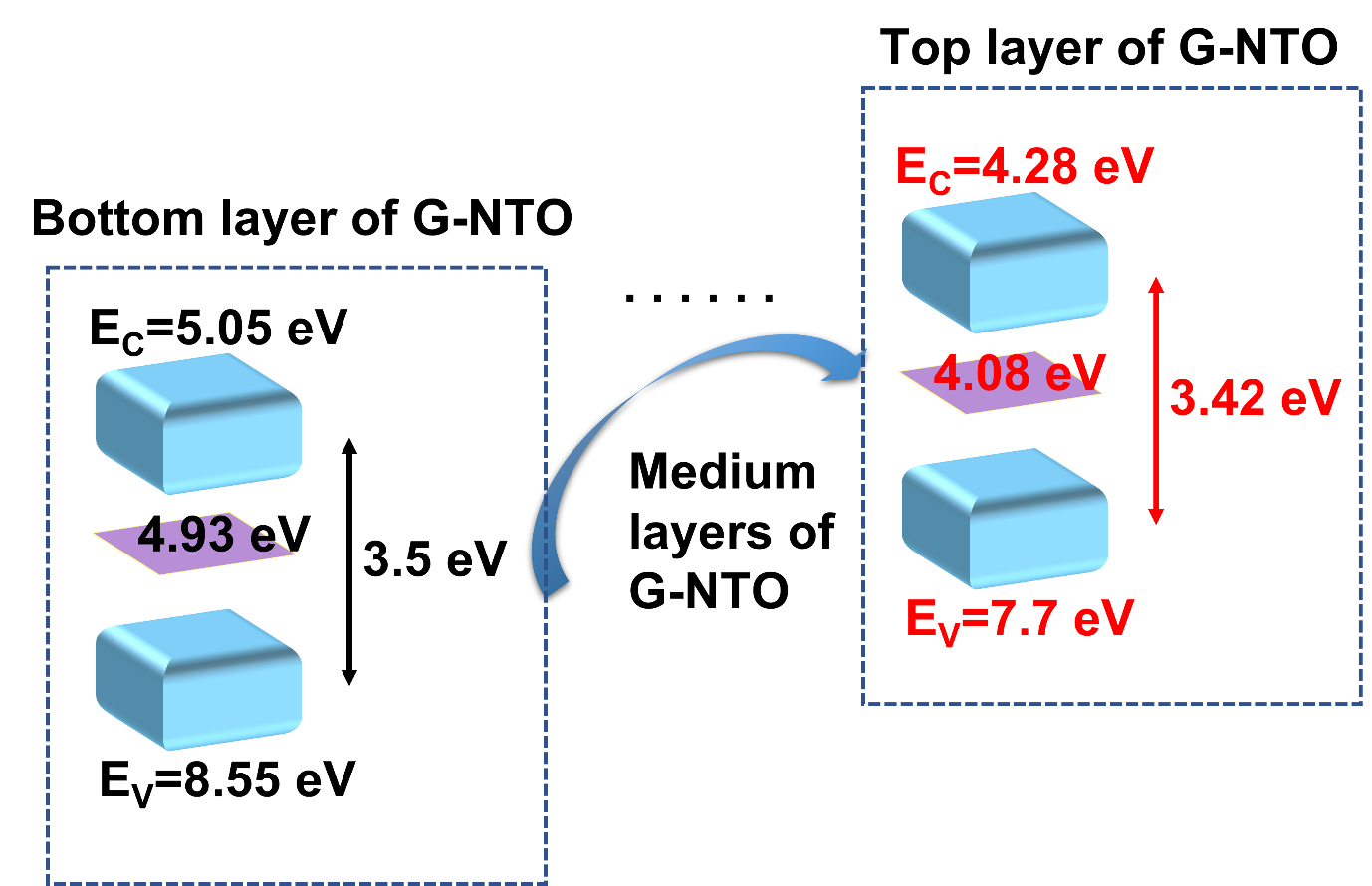


Fig. S12. Schematic diagram illustrating the range of band position regulation by G-NTO.


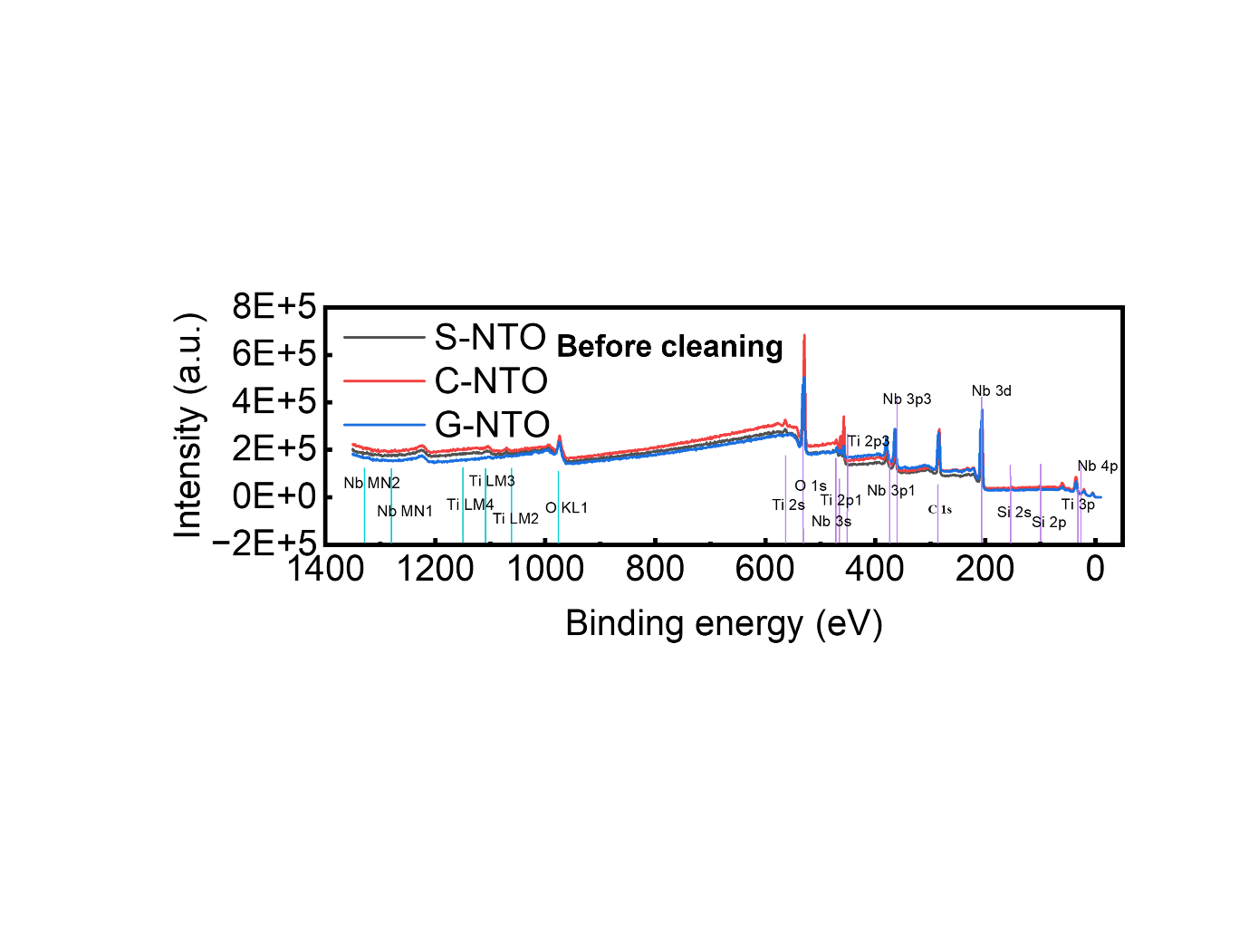


Fig. S13. XPS surveys of S-NTO, C-NTO and G-NTO films before cleaning.


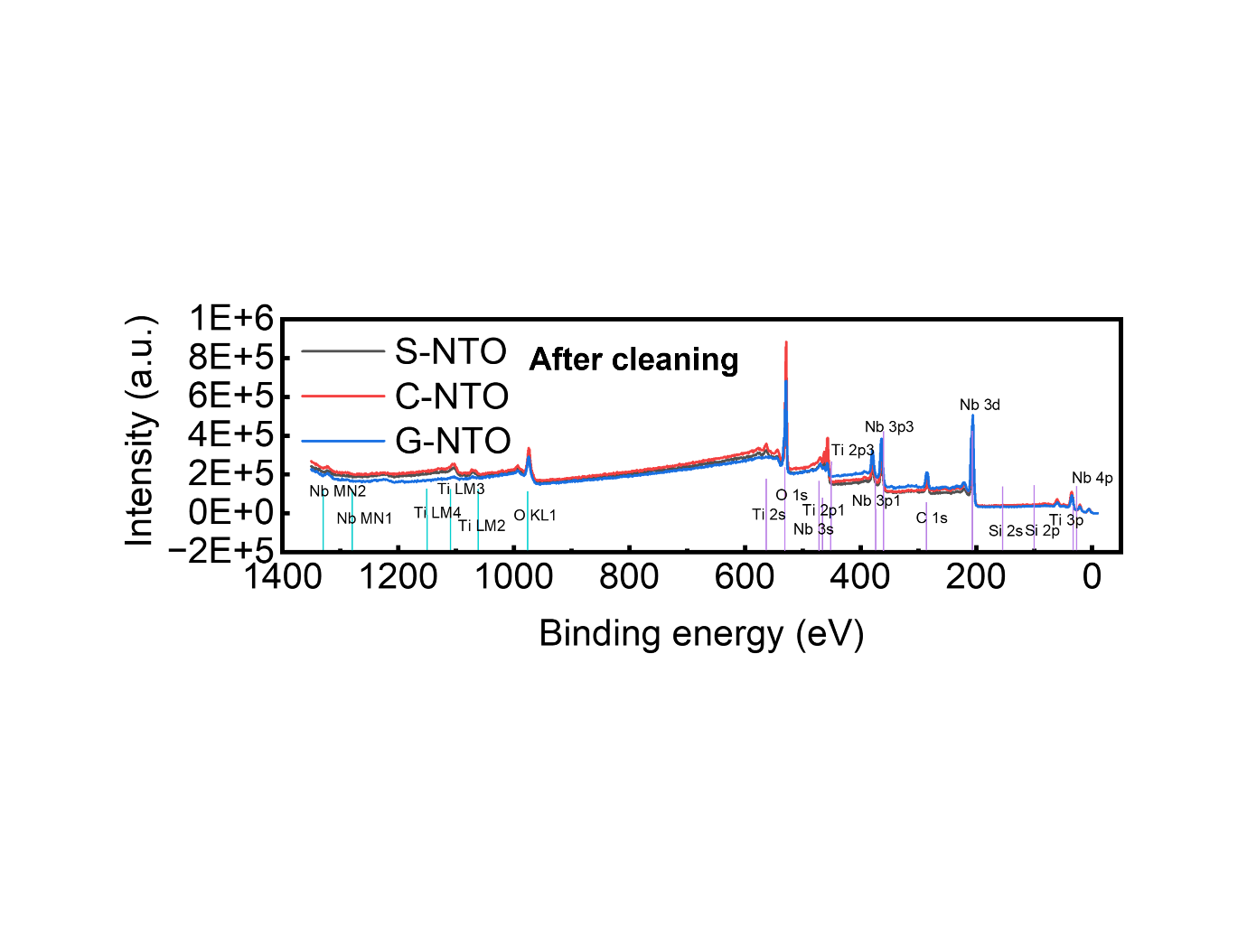


Fig. S14. XPS surveys of S-NTO, C-NTO and G-NTO films after cleaning.


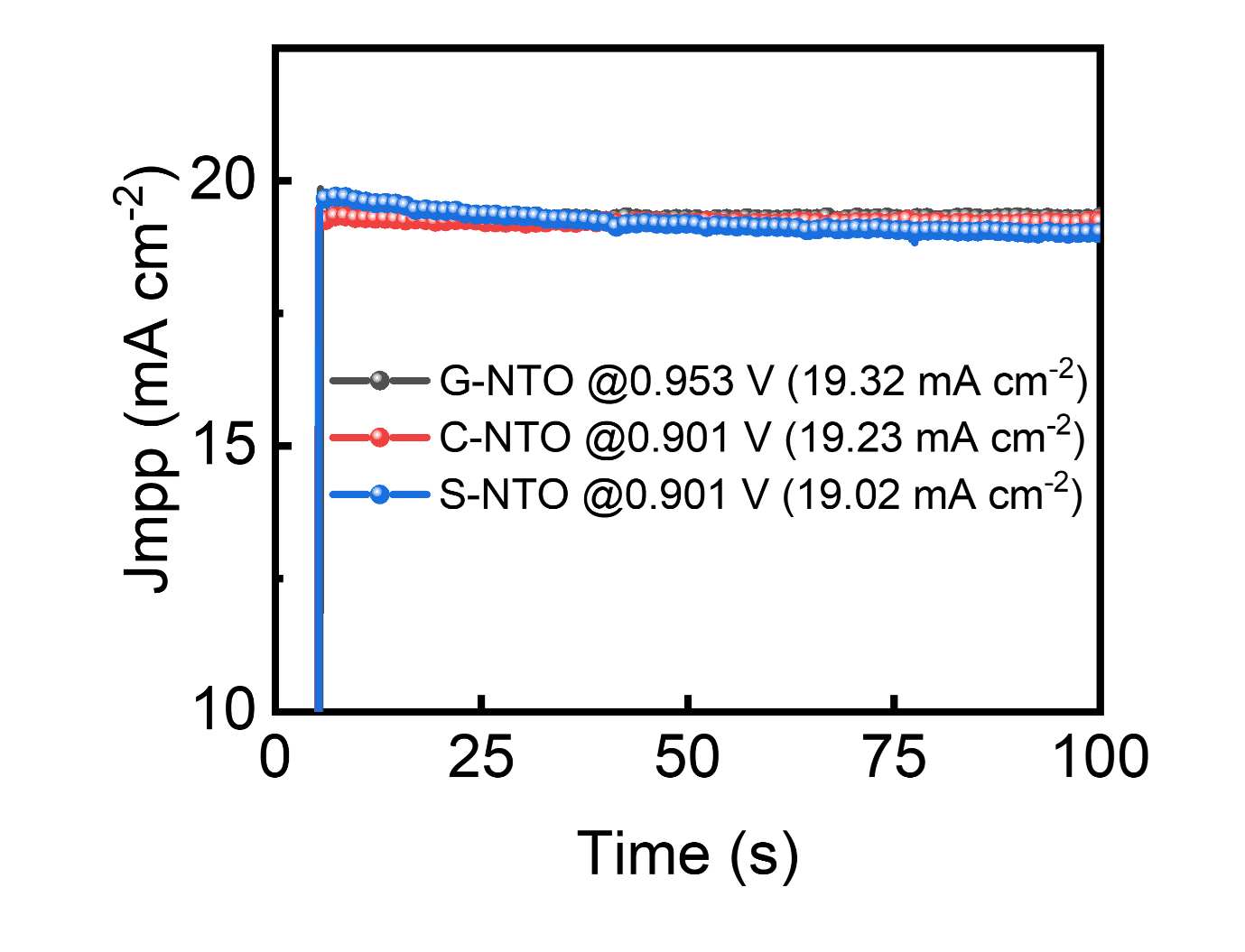


Fig. S15. Maximum power point (MPP) test results of the PSCs based on different NTO ETLs.


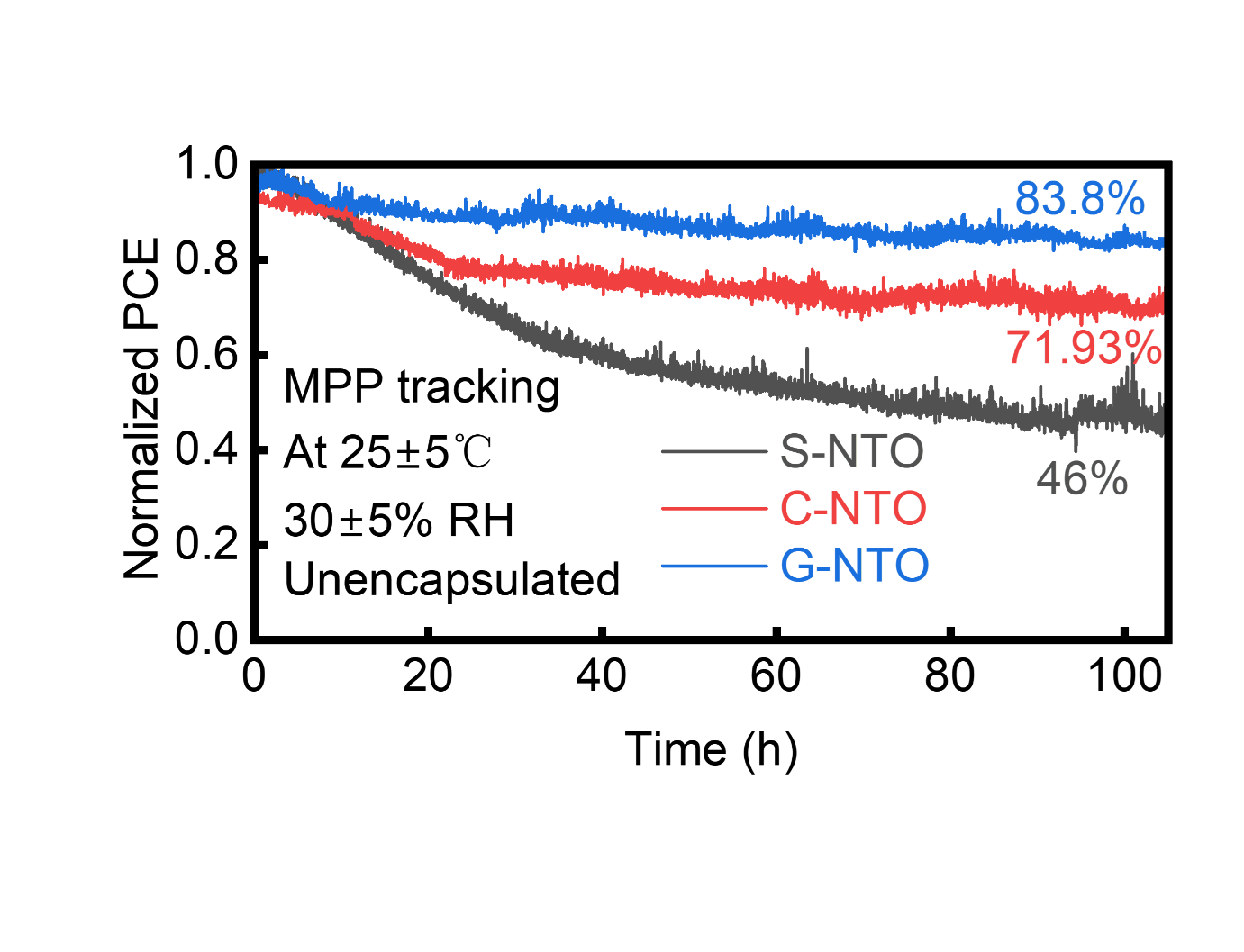


Fig. S16. The MPP tracking of unencapsulated devices measured in ambient conditions (25±5 ℃ with a relative humidity of 30±5 %, continuous light illumination).


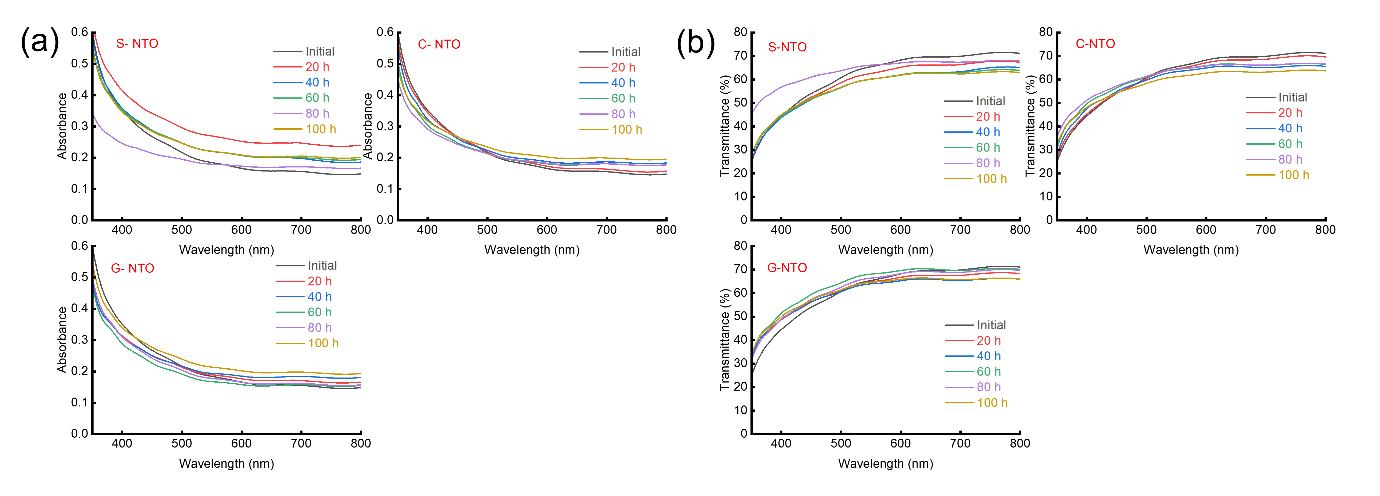


Fig. S17. Stability evolution of optical properties of S-NTO, C-NTO, and G-NTO films under 85 ℃-85% RH test for 100 hours.


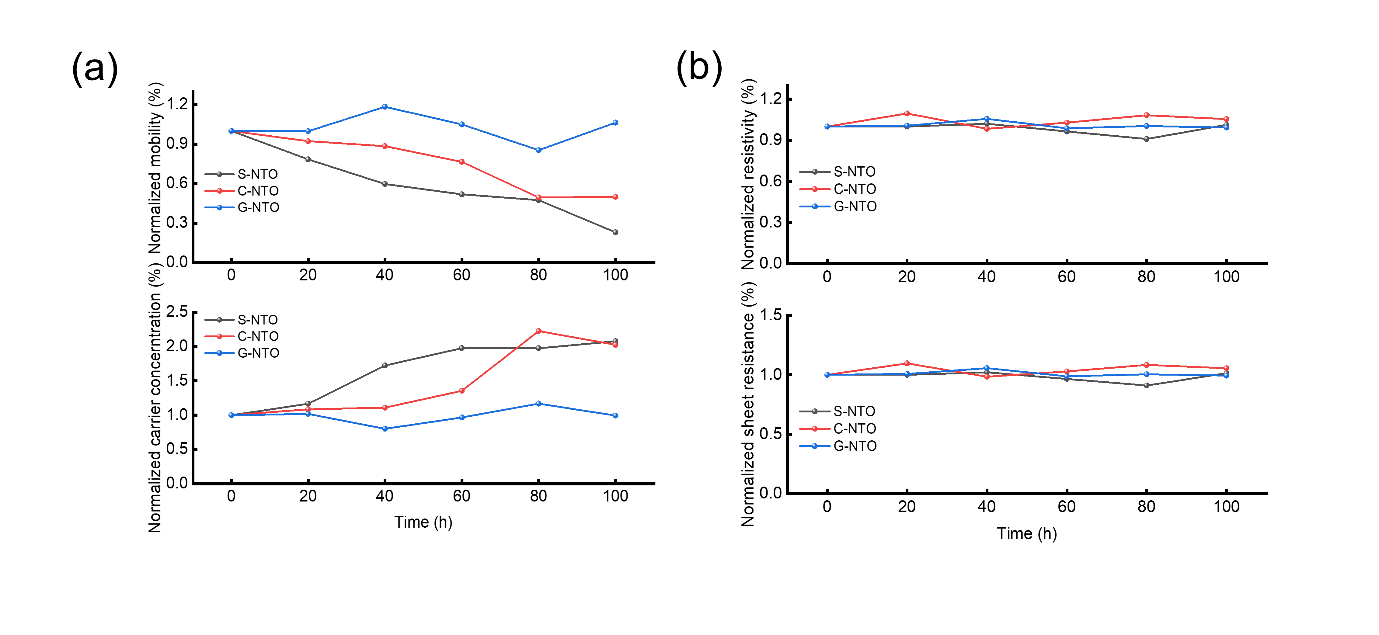


Fig. S18. Stability evolution of electrical properties of S-NTO, C-NTO, and G-NTO films under 85 ℃-85% RH test for 100 hours.

Table S1. Water contact angles of C-NTO films according to RF power applied to theNb_2_O_5_ target.

| Samples | Contact angle without  UV OT | Contact angle with  UV OT |
| --- | --- | --- |
| 0-NTO | 56.97° | 8.53° |
| 50-NTO | 57.36° | 9.33° |
| 75-NTO | 54.83° | 9.81° |
| 100-NTO | 56.76° | 10.01° |
| 125-NTO | 58.41° | 10.81° |
| 150-NTO | 64.72° | 11.00° |

Table S2. Water contact angles of NG-NTO and WG-NTO films

| Samples | Contact angle without UV OT | Contact angle with  UV OT |
| --- | --- | --- |
| NG-NTO | 55.43° | 9.35° |
| WG-NTO | 54.02° | 11.81° |

Table S3. Comparison of the devices’ performance produced in this work and literature

| Materials | Methods | PCE (%) | Reference |
| --- | --- | --- | --- |
| TiO_2_/Nb_2_O_5_ | Spin coating | 19.11 | [7] |
| Mg-doped SnO_2_ | Magnetron sputtering | 19.55 | [9] |
| Nb-doped TiO_2_ | Hydrothermal synthesis | 15.70 | [21] |
| Nb-doped TiO_2_ | Hydrolysis−pyrolysis | 18.88 | [22] |
| Nb_2_O_5_ | Magnetron sputtering | 5.74 | [26] |
| Nb_2_O_5_ | Magnetron sputtering | 17.10 | [28] |
| SnO_2_ | Hollow cathode gas flow sputtering | 13.93 | [30] |
| W-doped TiO_2_ | Solution-processed | 18.85 | [32] |
| Nb-doped TiO_2-x_ | Magnetron sputtering | 12.50 | [34] |
| Nb-doped TiO_x_ | Magnetron sputtering | 19.90 | This work |
